# Developing evidence‐based, cost‐effective P4 cancer medicine for driving innovation in prevention, therapeutics, patient care and reducing healthcare inequalities: Proceedings from a conference organised by the Pontifical Academy of Sciences and the European Academy of Cancer Sciences, held in Vatican City, May 22–23, 2025

**DOI:** 10.1002/1878-0261.70179

**Published:** 2025-12-15

**Authors:** Ulrik Ringborg, Joachim von Braun, Julio Celis, Anton Berns, Michael Baumann, Tit Albreht, Nancy Abou‐Zeid, Vanderlei Bagnato, Christian Brandts, Chien‐Jen Chen, Massimiliano di Pietro, Manjit Dosanjh, Thomas Dubois, Alexander Eggermont, Angelika Eggert, Ingemar Ernberg, Sara Faithfull, Johannes Förner, Stefan Fröhling, Manuel Heitor, Leroy Hood, Wei Jiang, Bengt Jönsson, Ravi Kannan, Maria Leptin, Su Li, Peter Lindgren, Douglas Lowy, Jun Ma, Alex Markham, Péter Nagy, Simon Oberst, M. Iqbal Parker, Danielle Rodin, Kevin Ryan, Joachim Schüz, Richard Sullivan, Josep Tabernero, Peter Turkson, Oliver Várhelyi, Harold Varmus, Chijie Wang, Elisabete Weiderpass, Nils Wilking

**Affiliations:** ^1^ European Academy of Cancer Sciences Stockholm Sweden; ^2^ Cancer Center Karolinska Karolinska University Hospital Stockholm Sweden; ^3^ Pontifical Academy of Sciences Vatican City Italy; ^4^ Bonn University Germany; ^5^ The Netherlands Cancer Institute Amsterdam The Netherlands; ^6^ German Cancer Research Center Heidelberg Germany; ^7^ National Institute of Public Health Ljubljana Slovenia; ^8^ Fondation ARC Villejuif Cedex France; ^9^ University of Sao Paulo Brazil; ^10^ Texas A&M University College Station TX USA; ^11^ UCT University Cancer Center, Frankfurt University Hospital Frankfurt Germany; ^12^ Academia Sinica Taipei Taiwan; ^13^ Early Cancer Institute University of Cambridge UK; ^14^ University of Oxford UK; ^15^ INCa, National Cancer Institute Boulogne Billancourt France; ^16^ University Medical Center Utrecht & Princess Máxima Center for Pediatric Oncology The Netherlands; ^17^ Charité Berlin Germany; ^18^ Karolinska Institutet Stockholm Sweden; ^19^ Trinity College Dublin The University of Dublin Ireland; ^20^ Patient Advisory Council of the German Cancer Research Center (DKFZ) and NCT Patient Research Council Heidelberg Germany; ^21^ Centre for Innovation, Tech. & Policy Research, IN+@IS Tecnico University of Lisbon Portugal; ^22^ Phenome Health and the Institute of Systems Biology Seattle WA USA; ^23^ Buck Institute for Research on Aging Novato CA USA; ^24^ Department of Radiation Oncology, State Key Laboratory of Oncology in South China, Guangdong Key Laboratory of Nasopharyngeal Carcinoma Diagnosis and Therapy, Guangdong Provincial Clinical Research Center for Cancer Sun Yat‐sen University Cancer Center Guangzhou China; ^25^ Stockholm School of Economics Sweden; ^26^ Cachar Cancer Hospital and Research Centre (CCHRC) India; ^27^ European Research Council Brussels Belgium; ^28^ Department of Clinical Research, Sun Yat‐sen University Cancer Center, State Key Laboratory of Oncology in South China Collaborative Innovation Center for Cancer Medicine Guangzhou China; ^29^ The Swedish Institute for Health Economics Stockholm Sweden; ^30^ National Cancer Institute Bethesda MD USA; ^31^ University of Leeds UK; ^32^ Department of Molecular Immunology and Toxicology and the National Tumor Biology Laboratory National Institute of Oncology Budapest Hungary; ^33^ Organisation of European Cancer Institutes, OECI Brussels Belgium; ^34^ University of Cape Town Faculty of Health Sciences South Africa; ^35^ Princess Margaret Cancer Center Toronto Canada; ^36^ Molecular Oncology Cambridge UK; ^37^ Cancer Research UK Scotland Institute Glasgow UK; ^38^ School of Cancer Sciences University of Glasgow UK; ^39^ International Agency for Research on Cancer Lyon France; ^40^ Institute of Cancer Policy, King's College London UK; ^41^ Vall d'Hebron Institute of Oncology Barcelona Spain; ^42^ EU Commissioner for Health and Animal Welfare Brussels Belgium; ^43^ Weill Cornell Medicine New York USA

**Keywords:** cancer prevention, cancer therapeutics/care, Comprehensive Cancer Center, inequalities, science policy, structuring translational cancer research

## Abstract

The cancer problem is expanding, particularly in low‐ and middle‐income countries (LMICs). Preventive measures can reduce the incidence by 40–50%, and cure rates have increased during the past decades in a number of cancers. However, optimizing prevention programmes and increasing cure rates of cancer remain significant research challenges. The main focus of the conference was on P4 Cancer Medicine (Predictive, Preventive, Personalized and Participatory), a comprehensive strategy encompassing Health‐Related Quality of Life (HRQoL) research, aiming to enhance the well‐being of patients and individuals at risk. Addressing the cancer problem requires two key elements: translational cancer research and the development of relevant infrastructures. A Comprehensive Cancer Centre (CCC) acts as an innovation hub by integrating high‐quality, multidisciplinary therapy and care, with healthcare‐dependent prevention, research, and education. The United States has been at the forefront, providing quality‐assured CCCs and the Cancer Moonshot for strategic cancer research. The EU has followed with the European Research Council for basic research, the European Innovation Council to boost disruptive innovation, and two EU initiatives on cancer, Europe's Beating Cancer Plan (EBCP) and the Mission on Cancer. The increasing complexity of cancer biology and technologies presents both a research challenge and a healthcare demand. For most patients, a CCC is not available. A critical discussion focused on quality assurance of healthcare outside the catchment area of a CCC and involving patients in clinical research. The strategic deployment of resources to support collective healthcare efforts and research aimed at reducing the cancer problem was discussed with representatives from the United States, EU, Africa, China, India and Taiwan. Analyses of translational cancer research have revealed important gaps in implementing innovations, assessment of clinical effectiveness, HRQoL, outcome and health economics research. The increased release of new anticancer agents over the last 25 years, accompanied by insufficient information on clinical benefits, presents both an economic and ethical problem. Direct healthcare costs have increased due to expenses for anticancer agents for the treatment of patients with incurable diseases. Evidence‐based treatment based on HRQoL research is an unmet need. Basic/preclinical research aimed at increasing the cure rate should identify new, broader targets for therapy and develop extended diagnostic technologies for stratifying patients, to inform innovative clinical trials. Present research strategies convert cancer to a chronic disease, a growing burden for the healthcare systems. The increasing complexity of cancer biology and technology, the growing need for translational cancer research, and the demand for supporting infrastructures underscore the importance of international collaborations between CCCs. However, funding for cancer research is not currently aligned to reduce the cancer problem. While public funding for cancer research doubled between 2005 and 2024, the pharmaceutical industry's spending on cancer research increased tenfold. Increasing funding by public and non‐profit funding organizations is mandatory. Education is another significant need, but it is currently fragmented and underfunded. The last session of the conference summarized the strategies in a Statement with a strong emphasis on global collaboration addressing the growing cancer burden and pronounced inequalities. Expanding partnerships and fostering innovative, multidisciplinary approaches to cancer prevention, therapeutics/care, as well as research, are not just urgent but essential steps towards reducing incidence, increasing cure rates and enhancing the well‐being of cancer patients. Data‐driven cancer medicine is currently under development, and modern communication technologies for diagnostics may facilitate interactions across geographical distances. A global cancer research agenda can become a model of solidarity, sustainability, and ethical responsibility.

## Graphical abstract

The cancer problem is increasing globally with projections up to the year 2050 showing unfavourable outcomes in terms of incidence and cancer‐related deaths. The main challenges are prevention, improved therapeutics resulting in increased cure rates and enhanced health‐related quality of life. These require strong translational cancer research with Comprehensive Cancer Centers serving as the main infrastructure. Translational cancer research is defined as a coherent cancer research continuum: from basic/preclinical research to prevention/clinical research, and further to outcomes and health economics research. The core concept in addressing the cancer problem is the development of P4 Cancer Medicine.

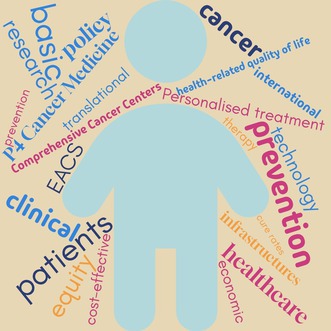

**Abbreviations**
AI: Artificial intelligence
CCC: Comprehensive Cancer Center
CCE: Cancer Core Europe
CPE: Cancer Prevention Europe
DKFZ: Deutsches Krebsforschungszentrum
EACS: European Academy of Cancer Sciences
EBCP: Europe's Beating Cancer Plan
EBV: Epstein–Barr virus
EIC: European Innovation Council
EORTC: European Organisation for Research and Treatment of Cancer
ERC: European Research Council
GDP: Gross Domestic Product
HBV: Hepatitis B virus
HCV: Hepatitis C virus
HICs: High‐Income Countries
HPV: Human Papilloma virus
LIMCs: Low‐ and Middle‐ Income Countries
MSCA: Marie Skłodowska‐Curie Actions
MTB: Molecular Tumour Board
NCI: National Cancer Institute
NCT: National Centre for Tumour Diseases
NGO: Non‐Governmental Organization
OECI: Organisation of European Cancer Institutes
PAS: Pontifical Academy of Sciences
PROMS: Patient‐Reported Outcome Measures
RWD: Real World Data
RWE: Real World Evidence
SME: Small and Medium‐sized Enterprise

## 1 Introduction

The escalating global cancer crisis, in tandem with the burgeoning field of cancer biology and technology research, has spurred the development of strategies to mitigate this issue in the late 20th century. The United States, a pioneering force in this field, has taken the lead with the establishment of Comprehensive Cancer Centres (CCCs). However, the translation of complex basic and preclinical research into clinical application, is a formidable although intellectually stimulating challenge, posing a significant difficulty in predicting outcomes. It was in response to this challenge that the concept of translational cancer research was born.

In Europe, strategic planning began in 2000 when the EU Commissioner for Research and Innovation, Philippe Busquin, initiated an EU project to analyse the fragmentation of European cancer research. Articulating the needs for a quality programme ensuring CCCs and how to structure European translational cancer research was the primary goal [1]. In 2009, the European Academy of Cancer Sciences (EACS) was established to help unify the European cancer community [2]. European activities were also stimulated by the launch of the Cancer Moonshot in the United States in 2016 [3].

In 2017, the Pontifical Academy of Sciences (PAS) of the Vatican and the EACS began a close interaction. Pope Francis had a strong agenda for decreasing inequalities whereas the EACS explored strategies aiming at mission‐oriented cancer research [4, 5]. A first conference was organized in Vatican City in 2018 on the concept of mission‐oriented cancer research [6, 7]. The conference contributed to the decisions by the European Commission to launch Europe's Beating Cancer Plan (EBCP) and the EU Mission on Cancer under respectively the EU4Health Programme and Horizon Europe Framework Programme for Research and Innovation, which started in 2021 [2]. A second conference was organized in 2023 to follow up on the implementation of the two EU initiatives with a focus on innovation and the role of research to mitigate inequalities [8]. This year, a third conference was organized to evaluate the outcomes of both EU initiatives, as well as international developments.

The agenda for the conference was designed to address the globally growing cancer problem. It underscored the crucial need for global strategies and the potential of translational cancer research to innovate in prevention, therapeutics and care with the aim of decreasing inequalities. As in previous conferences, PAS and EACS invited representatives from other continents to discuss global strategies and, at the same time, receive a critical opinion on the EU initiatives.

**Fig. 1:**
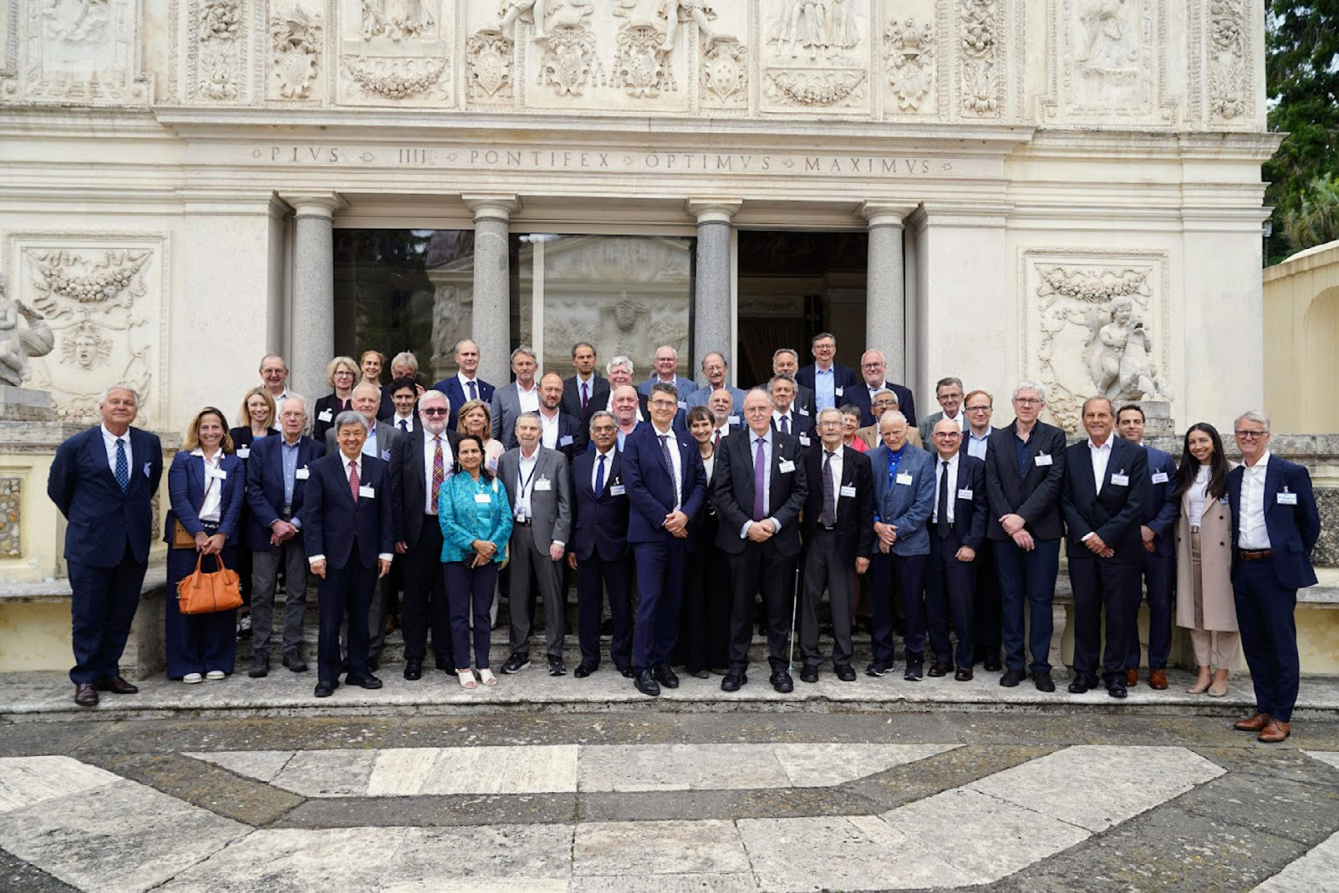
Photograph of conference participants. Photo by Gabriella C. Marino, PAS.

Participants were welcomed to the conference by Joachim von Braun, President of the Pontifical Academy of Sciences, and Michael Baumann, President of the European Academy of Cancer Sciences (Fig. 1).

In a highly anticipated keynote lecture **Harold Eliot Varmus**, former Director of the National Institutes of Health and NCI, USA, discussed **Prospects for bringing the benefits of modern science and advances in the control of cancer to low‐ and middle‐income countries (LMICs)**.

In the past few decades, dramatic advances in the biological sciences, mostly in high‐income countries (HICs), have promoted remarkable progress towards understanding the mechanisms of carcinogenesis; identifying the genetic alterations that drive neoplasia; and developing new methods for detection, classification, and treatment of cancers (including targeted therapies and immunotherapies). However, many obstacles—political, economic and technical—have impeded the equitable deployment of these advances for the benefit of all people, especially poor people living in less affluent countries.

A variety of approaches may help to reduce such unfair disparities. These approaches include (i) the design of research programmes intended to improve cancer prevention and care in poor countries; (ii) steps that encourage the wider use of modern technologies—especially genomics and digital sciences—to enhance scientific and clinical work; (iii) collaborative projects that share expenses, expertise, and access to advanced knowledge; and (iv) policies that promote investments in science and health care.

The value of these approaches was illustrated by discussing several projects that aim to reduce the burden of diseases, including cancers, in poor countries. These examples illustrate the value of investments in medical research by governments, foundations and the commercial sector, as well as the benefits of collaborations among laboratories and nations. They include work undertaken by the Uganda Cancer Institute, the National Institutes of Health (e.g. the Centre for Global Health at the NCI, the Gates Foundation–the Grand Challenges in Global Health) and the World Health Organization's Science Council (e.g. reports on genomics and digital health).

By discussing such examples, he hoped to encourage more investment and collaboration at a time when scientific opportunities are often not matched by an appropriate spirit of international cooperation.

## 2 Session 1: The increasing cancer problem: initiatives in world regions

**Chair Maria Leptin**, President of the European Research Council, Brussels, Belgium

**Elisabete Weiderpass**, Director, International Agency for Research on Cancer (IARC), Lyon, France, presented an **overview of the increasing worldwide cancer problem and inequalities in prevention and care**, a comprehensive overview of the escalating global cancer burden, highlighting stark inequalities in cancer prevention, diagnosis, and treatment across regions. In addition, she presented recent evidence from IARC studies that underscore the substantial potential of preventive strategies in reducing cancer incidence and mortality worldwide.

In 2022, an estimated 20 million new cancer cases and nearly 10 million cancer‐related deaths occurred worldwide, with projections indicating a 77% increase in incidence by 2050—disproportionately affecting LMICs. These trends call for urgent, cost‐effective, and equity‐focused interventions.

The presentation delved into major emerging challenges such as rising lung adenocarcinoma rates, especially among young women, and increasing colorectal cancer incidence in younger adults. It also addressed the projected 40% rise in breast cancer cases and deaths by 2050, particularly in low Human Development Index (HDI) countries. IARC's Mutographs Project offers insights into cancer causation, including the role of colibactin‐producing bacteria in early‐onset colorectal cancer.

Preventive measures—such as tobacco control, human papilloma virus (HPV) vaccination, and lifestyle changes—are shown to be highly effective. Elisabete Weiderpass highlighted IARC's support for WHO global initiatives, including cervical and breast cancer strategies, and the importance of addressing social determinants of health to ensure equitable access to care.

Ultimately, the presentation called for urgent, evidence‐based action—through targeted research, innovation, and international collaboration—to reduce global cancer disparities and improve outcomes for all populations, particularly in resource‐limited settings.

*Where authors are identified as personnel of IARC/WHO, the authors alone are responsible for the views expressed in this article, and they do not necessarily represent the decisions, policy, or views of IARC/WHO*.

**Ulrik Ringborg**, Cancer Center Karolinska, Stockholm, Sweden, discussed the **development of evidence‐based and cost‐effective P4 Cancer Medicine for** the **innovation of prevention and therapeutics/care and reduction of inequalities**. The increasing cancer problem worldwide must be counteracted by addressing the following challenges: decrease incidence; increase cure rate; improve survival and patients' Health‐Related Quality of Life (HRQoL). Cancer incidence can be significantly reduced by implementing known preventive measures whereas increasing cure rates poses a demanding strategic research challenge. Present overall strategy converts cancer to a chronic disease group, a future problem for the healthcare systems. Improvement of survival for patients with incurable disease is another goal. For the latter it is crucial that HRQoL research is given more visibility and support to enhance patient care.

**Fig. 2:**
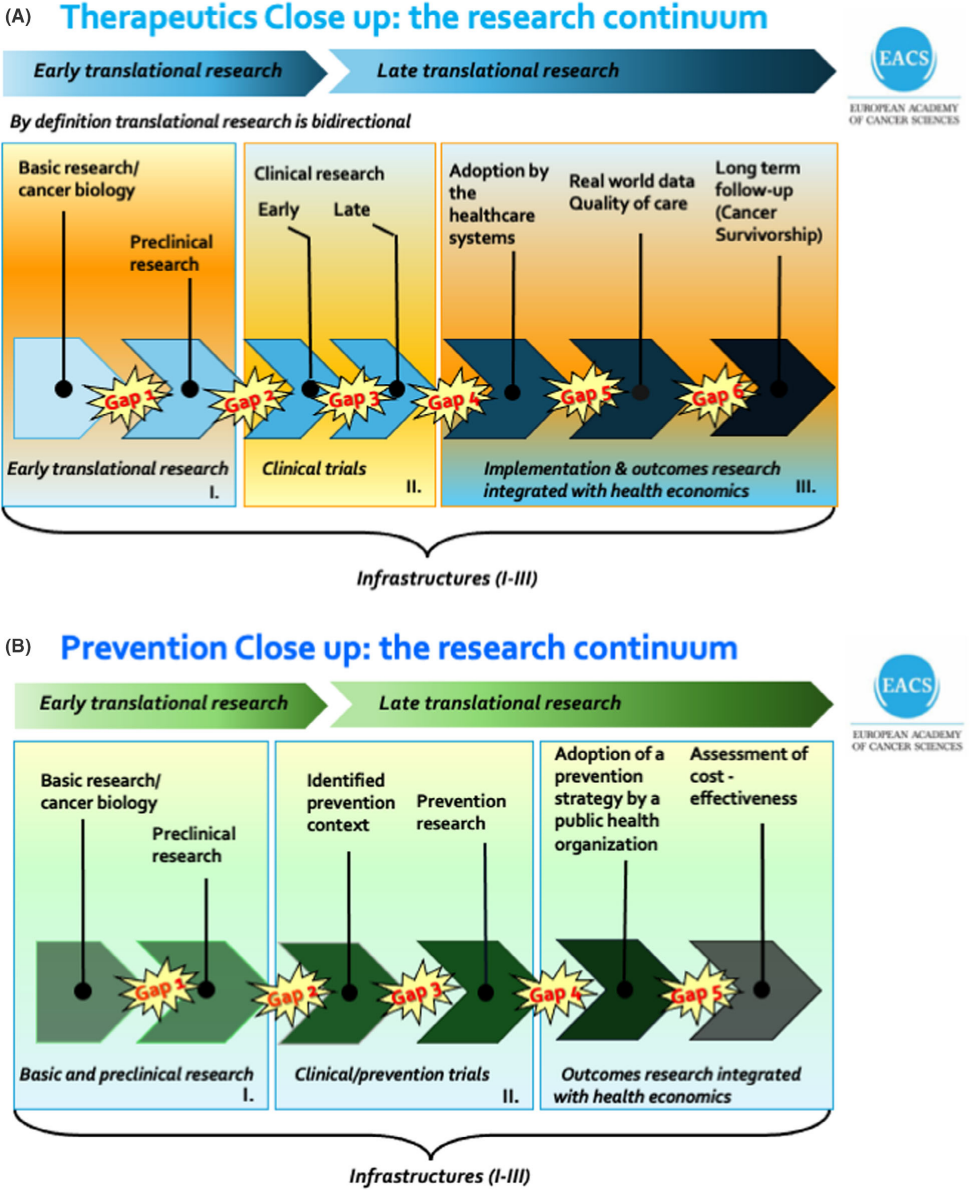
The cancer research continuum for therapeutics and prevention. An outline of the relevant modalities and gaps within the cancer research continuum for (A) cancer therapeutics and (B) for cancer prevention. Translational cancer research is defined as a coherent research continuum with bridged gaps, which often requires multidisciplinary research collaborations. Reverse translational research is increasing in importance when the research continuum is complete and coherent. Unexpected observations (examples, adverse reactions or resistance during treatment) give rise to new questions for basic/preclinical research.

Translational cancer research, mandatory to tackle the cancer problem, is defined as a coherent research continuum for cancer diagnostics, therapeutics, holistic care and prevention (Fig. 2). Present gaps should be bridged by improved multidisciplinary collaborations. The goal is P4 Cancer Medicine, which means that cancer medicine should be Predictive, Personalized, Preventive and Participatory, thereby optimizing the well‐being of patients [9].

The CCC integrates multidisciplinary care, research, education and, in several aspects also elements of cancer prevention. The CCC with integrated basic/preclinical research is the fundamental infrastructure for translational cancer research. Although an increasing number of quality‐assured CCCs is gradually established, most patients do not live in the catchment area of a CCC. A critical question is how to quality assure the healthcare and actively involve patients in clinical research in a defined outreach area associated with a CCC. This is crucial in offering all patients high‐quality and innovative therapeutics/care.

Suboptimal or missing elements in the cancer research continuum contribute to inequalities. Examples are: preclinical prevention and therapeutic research, structuring prevention/clinical trials for implementation, HRQoL, outcomes and health economics research.

Critical mass regarding patients, biological materials and technological resources is an increasing problem. Single CCCs often lack the critical mass to innovate translational cancer research. National and international collaborations between CCCs are therefore a must. Furthermore, funding mechanisms are not tuned to the goals of mission‐oriented cancer research: support for the establishment and sustainability of CCCs and support for translational research dealing with all aspects of prevention and therapeutics/care are needed. Education is an additional big challenge.

A long‐term goal for translational cancer research is to boost innovation and decrease inequalities by establishing evidence‐based cancer prevention and therapeutics/care through improved national and international CCC collaborations, together with resource sharing.

**Douglas Lowy**, NCI, USA, spoke on **Addressing health disparities with the U.S. Cancer Moonshot and beyond**. The presentation began by noting that the USA Cancer Moonshot and the EBCP share two critical goals: (a) improving the options for preventing, screening, detecting, and treating cancer through research; (b) narrowing and eliminating the disparities in the unequal cancer incidence and outcomes within the populations of EU countries and the USA that are mainly attributable to unequal access to cancer care and control. Beyond this EU‐centric and USA‐centric framework, cancer disparities between HICs, such as the EU and United States, and LMICs are generally much greater than those within HICs. Furthermore, it is predicted that most of the increase in cancer incidence and mortality over the next 20 years will occur in LMICs. It is therefore important to consider approaches that can reduce the global disparities in cancer in addition to those within HICs. Although several population‐wide interventions can be envisioned, two were highlighted. The first is to even more strongly promote efforts to reduce tobacco consumption worldwide, as this carcinogen is responsible for around one‐third of all cancers, in addition to being a major contributor to serious cardiovascular and pulmonary disease. The second is to reduce the risk of cervical cancer, which is one of the most common female cancers in LMICs, through HPV vaccination* and cervical cancer screening. The overall potential for technology to help reduce cancer disparities was also noted. For this to happen, it is important from the outset to consider the impact of new technology on cancer disparities.

*Disclosure: The author is one of the developers of the technology that underlies the HPV vaccine.

**Maria Leptin**, President, European Research Council (ERC), Brussels, Belgium, delivered a presentation on **Driving breakthroughs in cancer research: the role of frontier science and data integration**. The landscape of cancer research is undergoing rapid transformation, marked by significant advances and coordinated global initiatives. Recent efforts such as EBCP and the EU Mission on Cancer reflect robust European frameworks designed to enhance research and patient care, while global programmes like the US Cancer Moonshot and China's Healthy Action Plan underscore a shared worldwide commitment to reducing cancer mortality. A key development in this context is the concept of P4 Medicine—Predictive, Preventive, Personalized and Participatory—together with translational research approaches through CCCs, which integrate patient care, research and education. These coordinated efforts have helped accelerate the translation of scientific discoveries into clinical applications.

To advance progress in cancer research, it is crucial to maintain a delicate balance between structured, top‐down strategies and curiosity‐driven, bottom‐up research. While coordinated efforts provide strategic direction and enable the rapid application of discoveries, it is investigator‐led frontier research—such as that championed by the ERC—that often generates transformative breakthroughs. Innovations like mRNA vaccines and CRISPR‐Cas gene editing arose from such curiosity‐driven exploration, underscoring its indispensable role in advancing cancer research. Yet, this type of research often receives insufficient policy attention, as pressures for immediate results tend to sideline longer‐term exploratory projects. Supporting bottom‐up initiatives should not be viewed as competing with structured programmes but as a vital complement, forming the foundation for future applied innovations.

The next decades are expected to bring giant leaps in cancer research through the combination of AI technologies and high‐quality, large‐scale datasets. Although Europe possesses rich data resources, fragmentation, siloing and regulatory hurdles currently limit access and sharing. Disparate systems and varying interpretations of GDPR across countries create additional barriers to cross‐border collaboration. To overcome these challenges, this talk advocates for the creation of centralized European data repositories, harmonized regulations, and dedicated financial support for data infrastructure. Incentivizing data sharing—including from private companies—and fostering open‐source initiatives will be essential for unlocking the potential of AI and large‐scale analyses in healthcare.

**Joanna Drake**, Deputy Director‐General of the European Commission's Directorate‐General for Research and Innovation and Cancer Mission Manager, Brussels, Belgium, reviewed the **EU Cancer Mission** in an online presentation.

The RTD Deputy Director‐General, Ms Joanna Drake, outlined the EU Cancer Mission's central goal: improving the lives of 3 million people by 2030 through prevention, cure, and improving the quality of life of cancer patients and their families. The Mission works in close synergy with EBCP and regional initiatives to align cancer research, innovation, and care across the EU. Overall, the Cancer Mission portfolio is structured according to its four objectives: understanding, prevention and early detection, diagnosis and treatment, and quality of life.

Through innovation—one of its five transversal priorities—the EU Cancer Mission contributes to a pan‐European innovation ecosystem to beat cancer. It does this by bringing together Member States and regions, researchers, stakeholders from different sectors and disciplines, investors and citizens. Three examples:

Through Horizon Europe programmes like the ERC and EIC, with €380 M invested in frontier research and €590 M backing start‐ups and SMEs. Second, by scaling up successful national and regional pilots that provide tailored services to implement cancer screening programmes in countries like Latvia, Slovakia, Slovenia and Luxemburg using the Technical Support Instrument managed by DG REFORM. Third by a pan‐European innovation ecosystem through collaboration with the EIT‐Health Knowledge and Innovation Community and the upscaling of national and regional research and care pilots.

Two flagship projects were highlighted: UNCAN.eu—a federated cancer research data hub—and a comprehensive cancer infrastructure network to ensure that 90% of the EU population has access to cancer prevention and care. UNCAN.eu will integrate large‐scale patient and research data using platforms like EOSC4Cancer, CanServe and EUCAIM within the European Health Data Space policy framework.

Also, National Cancer Mission Hubs are being launched by the ECHoS project to align EU and Member State priorities and foster long‐term health‐research policy integration, starting with the launch of the Polish Cancer Mission Hub in Warsaw last May. Ms Drake ended her intervention by calling for the Mission to attract third‐party investments and stimulate international cooperation, highlighting partnering with the United States and Japan.

**Olivér Várhelyi**, Commissioner for Health and Animal Welfare, European Commission, Brussels, Belgium, reported on **Europe's Beating Cancer Plan (EBCP)** (video message). The Commissioner appreciated the election of Pope Leo XIV as a deeply profound moment for Catholics in Europe and around the world. The Holy See has a special role to play in bringing people together to address the major issues of our time, including our health. The Commissioner is looking forward to continuing to work closely together with the Catholic Church.

Cancer continues to present an enormous challenge with 2.7 million new cases per year just in the European Union. In the EU, the response is guided by EBCP which covers the whole pathway of the disease: from prevention, early detection, diagnosis and treatment to the quality of life of patients, survivors and their families. Since the launch of the EBCP in 2021, 90 percent of its initiatives are either ongoing or concluded.

Research, innovation and new technologies are cross‐cutting topics in EBCP. Research is vital to tackling cancer and for turning personalised medicine into reality. At the same time, personalised medicine provides the data, methodologies and patient focus that can take research forward. The European Health Data Space is supporting the seamless integration of health data into research.

Horizon Europe, the EU's Research and Innovation Framework Programme (2021–2027) supports bringing concrete results on cancer. Since its launch in 2021, the EU Cancer Mission has committed nearly EUR 500 million to 61 research and innovation projects, in close collaboration with the EBCP. This complements the funding committed for the activities of the EBCP of more than EUR 394 million from the EU4Health Programme.

**Chijie Wang, Wei Jiang, Su Li** and **Jun Ma**, Sun Yat‐sen University Cancer Centre, Guangzhou, China, informed on **China policies for Cancer Research, Healthcare and Prevention: Structuring translational research to increase innovation and reduce inequalities**. Cancer has become one of the leading health threats to the Chinese population. In 2022, over 4.8 million new cancer cases and approximately 2.5 million cancer‐related deaths occurred in China, with lung and colorectal cancers among the most prevalent. The economic burden resulting from treatment costs and productivity loss is substantial. In response, China has implemented a series of policy measures and established a translational research framework to improve early detection, drug development and personalized treatment.

In early cancer screening, China has steadily expanded nationwide programmes since 2005, focusing on high‐incidence cancers such as lung, oesophageal, gastric, colorectal, breast and cervical cancer. By 2018, upper gastrointestinal cancer screening had reached over 2.16 million individuals, identifying 34 607 cancer cases, with a detection rate of 2.05% and an early diagnosis rate exceeding 70%. The early diagnosis rate for liver cancer also rose markedly, from 66% in 2008 to 97% in 2022. In addition, China has issued early detection and treatment guidelines for multiple cancer types and supports the establishment of dedicated screening centres and training programmes at the county hospital level.

In drug development, China has established an accelerated review and approval mechanism, leading to the approval of 158 new anti‐tumour drugs between 2015 and 2024, including 72 domestically developed originator drugs. Conditional approvals have shortened the time to market. Regulatory reforms have also standardized the use of single‐arm trials, limiting them to refractory diseases and requiring survival‐related endpoints, with independent review committees minimizing assessment bias. For combination therapies, approval must be supported by mechanistic studies and evidence of contribution from each component. The overall regulatory approach is guided by clinical value, prioritizing unmet medical needs and encouraging adaptive trial designs.

China is also advancing personalized cancer care by promoting genetic testing and tiered treatment models. EGFR mutation testing in non‐small cell lung cancer has reached almost 50%, and in some regions, genetic tests are now covered by health insurance. Meanwhile, about 82% of hospitals have implemented bidirectional referral systems to support integrated care.

Despite notable progress, challenges remain—including prolonged timelines for translating research into clinical practice, limited primary care capacity, and regional disparities. Looking ahead, it will be important to strengthen policy guidance, enhance coordination mechanisms, invest in primary care, expand telemedicine, and establish a comprehensive, life‐course‐oriented cancer control system—contributing to the goals of the *Healthy China 2030* initiative.

**Chien‐Jen Chen**, Genomics Research Centre, Academia Sinica, Taipei, Taiwan, reported on the **elimination of cancers caused by bacteria and viruses in the Asia‐Pacific region**. The World Health Organization (WHO) proposed six targets for the global elimination of hepatitis B virus (HBV) and hepatitis C virus (HCV) to reduce the mortality from liver cancer in 2015. Many Asia‐Pacific countries have launched national programmes to eliminate chronic viral hepatitis. Taiwan launched the HBV vaccination programme in 1984, the blood and injection safety programme in 1980, the harm reduction programme for the people who inject drugs in 2000, as well as the HBV and HCV diagnosis and treatment programme in 2003. Both the incidence and mortality rates of liver cancer have decreased significantly in Taiwan. The liver cancer incidence and mortality rates in the Asia‐Pacific region will be significantly reduced if HBV and HCV are eliminated in an effective and efficient way.

*Helicobacter pylori* (HP) infection is an important risk predictor for gastric cancer in the Asia‐Pacific region. Programmes for detecting HP infection using endoscopy and biomarkers have been carried out in many Asia‐Pacific countries. Taiwan launched the national HP elimination programme in 2004 resulting in the continuous decrease in incidence and mortality rates of gastric cancer. The gastric cancer incidence and mortality rates in the Asia‐Pacific region are expected to reduce drastically through the screening and treatment of HP infection.

Cervical cancer is one of the most common cancers among women in Asia‐Pacific countries, but it is preventable, screenable, early detectable and treatable. Despite making progress towards the WHO 90–70–90 targets for cervical cancer elimination through HPV vaccination, screening and treatment, no Asia‐Pacific country has reached all 3 targets. The national Pap smear screening programme in Taiwan was launched in 1995, showing more than 70% reduction in cervical cancer incidence and mortality rates in the last 25 years. Despite the compelling efforts in cervical cancer prevention and control, there remains insufficient investment in preventive measures in Asia–Pacific countries.

The incidence of nasopharyngeal carcinoma (NPC) is extraordinarily high in southern China and southeastern Asia. Based on the long‐term follow‐up studies in Taiwan, Epstein–Barr virus (EBV) biomarkers have been found to be useful for predicting NPC risk. Tests of serum EBV antibody and plasma EBV DNA levels were found to have favourable performance characteristics and cost‐effectiveness in high‐risk populations. The NPC mortality will be significantly reduced if the EBV screening methods are effectively applied.

**M Iqbal Parker**, University of Cape Town, South Africa, discussed **Private versus public programmes for cancer research, prevention and patient support in South Africa**. Cancer is currently the fifth leading cause of death in Africa, with more than 1 million new cases and 700 000 cancer‐related deaths annually. Globally there are about 10 million cancer deaths per year with about 70% occurring in LMICs but the latter have < 5% of the total global resources for combating cancer. The 2040 forecast for Africa is a doubling of the incidence to 2 million per annum with an estimated 1.4 million deaths annually.

Due to factors such as inadequate research and diagnostic facilities, lack of cancer awareness, poor training of healthcare providers, and inadequate preventive, diagnostic, and therapeutic resources, cancer care remains inaccessible to many Africans. This disproportionately affects poorer populations, aggravating existing health disparities.

Although several countries are increasingly recognising the burden that cancer places on the health system the investment in resources to improve patient outcomes remains hopelessly inadequate. In most countries, including South Africa, the public health care system is vastly under‐resourced and poorly managed. Many new‐generation biopharmaceuticals are mostly not available at public hospitals. The private health care facilities, on the other hand, are well managed and well resourced, but not affordable to those without health insurance.

The Cancer Association of South Africa (CANSA) does not have any cancer treatment facilities, nor does it provide any cancer treatment in any form. CANSA provides away‐from‐home accommodation at Cancer Care Homes in the main metropolitan areas to patients undergoing cancer treatment at oncology clinics far from their homes. Guests generally stay for an average of 6 weeks and receive meals and transport to and from treatment centres.

Africa has the lowest Gross Domestic Expenditure on R&D (GERD) worldwide, with a Sub‐Saharan Africa average of 0.44% of GDP. In contrast, the region spends almost 4 times as much (1.7% of GDP) on military hardware. Countries in North Africa spend as much as 4% of GDP on military hardware. South Africa, Kenya, Egypt, and Morocco have Africa's highest GERD of 0.85%, 0.81%, 0.72% and 0.71% of the GDP, respectively.

Addressing challenges to ensure universal cancer equity in Africa will improve the chances of longer quality survival times for cancer patients. Improved awareness, education and research will contribute towards lowering the cancer incidence for groups that are at high risk of developing cancer and can promote equitable access to cancer care on the continent.

**R. Ravi Kannan**, Cachar Cancer Hospital and Research Centre, Silchar, India, delivered a presentation on **Promoting Equitable Cancer Care in India: Multi‐sectoral Roles and Collaborative Efforts**. Cancer poses a significant public health burden in India, with over 1.4 million new cases and 0.85 million deaths annually. Despite modest improvements in recent decades, stark inequities persist in cancer care access, particularly among socioeconomically disadvantaged and geographically remote populations. Challenges such as late diagnosis, treatment deferral and inadequate palliative care services continue to affect vulnerable communities. However, India has been witnessing the emergence of a robust, multisectoral response involving central and state governments, professional networks and non‐governmental organizations (NGOs), fostering a more equitable ecosystem for cancer prevention, diagnosis, treatment and follow‐up.

Several national initiatives underpin this transformation. The National Cancer Control Programme (NCCP), launched in 1975, has laid the foundation for systematic cancer prevention and care. It was further strengthened by the National Cancer Registry Programme and the later integration with the National Programme for Prevention and Control of Cancer, Diabetes, Cardiovascular Diseases and Stroke (NPCDCS) in 2008. More recently, the National Cancer Grid (NCG), a consortium of over 360 institutions, has been instrumental in standardizing care protocols and ensuring quality services across the country. The Ayushman Bharat scheme, particularly the Pradhan Mantri Jan Arogya Yojana (PMJAY), has significantly mitigated out‐of‐pocket expenditures by providing free cancer treatment to millions. Additionally, the Ayushman Bharat Digital Mission (ABDM) leverages digital infrastructure to improve care coordination and continuity.

NGOs play a critical role in bridging last‐mile gaps. They contribute across the spectrum—from screening and awareness to home‐based palliative care—demonstrating nimble, community‐driven innovation. Between 2022 and 2024, NGOs screened over a million women for cervical cancer, delivered home care to 20 000+ patients annually, and helped organize HPV vaccination campaigns in states like Sikkim and Punjab.

A powerful example of grassroots innovation is the Cachar Cancer Hospital and Research Centre in Assam, a community‐led initiative that addresses socio‐economic and infrastructural barriers to care. Through strategic cost‐reduction, pro‐poor branding, and technology‐enabled outreach, the hospital reduced treatment deferral from over 70% to under 30% over a 15‐year period.

This evolving multisectoral landscape in India illustrates that equitable cancer care is achievable through collaboration among governments, civil society and professional networks. Future progress demands integrated use of data, digital tools, community engagement and policy innovation, with a sharp focus on the needs of marginalized populations. The Indian model offers valuable lessons for other LMICs pursuing equity in cancer care.

### 2.1 Conclusions of presentations and discussions

Regarding strategic cancer research, the USA Moonshot is the first example, followed by the ERC for basic research of excellence and the two EU initiatives for translational research, EBCP and the Mission on Cancer.

Projections indicate a 77% increase in cancer incidence by 2050, with the greatest burden falling on LMICs. The growing disparities between HICs, such as the United States and EU, and LMICs highlight the urgent need for increased research investment and resource allocation in LMICs. Progress can only be achieved by the strategic deployment of resources to support collective healthcare efforts and research that reduces incidence and improves cure rates. A comprehensive strategy requires integrating curiosity‐driven basic and preclinical research with structured top‐down approaches in prevention, treatment and care, thereby ensuring a balanced and effective approach to cancer prevention and treatment.

Globally, prevention deserves greater attention as emphasised by the IARC. The NCI has focused on early detection through screening programmes, and EBCP has prevention high on the agenda. China is expanding its early detection programmes and Taiwan has successfully implemented initiatives targeting HBV and HCV elimination, Helicobacter pylori infection control for gastric cancer prevention and a national Pap smear screening programme to prevent cervical cancer. Nasopharyngeal carcinoma is highly prevalent in southern China and southeastern Asia. EBV biomarkers are proving valuable for identifying at‐risk populations.

China's accelerated drug review and approval system has enabled the approval of 158 new anticancer agents for personalized cancer treatment based on genetic testing which is truly impressive.

Africa faces severe limitations in research support, diagnostic capacity, prevention and treatment. Cancer care remains largely inaccessible to poorer populations, deepening existing health disparities. The private healthcare facilities, on the other hand, are well managed, but not affordable to those without health insurance which is a cause of empathy. The Cancer Association of South Africa (CANSA) provides some relief by offering accommodation in Cancer Care Homes for patients receiving treatment far from home in the main metropolitan areas. It is worth noting that Africa has the lowest Gross Domestic Expenditure on R&D worldwide.

India is developing a multisectoral activity involving several central and state governments, professional networks, and NGOs for cancer prevention, diagnosis, treatment, and follow‐up. A screening programme for cervical cancer, including HPV vaccination, and a home‐based palliative care programme are run by NGOs. Achieving equitable cancer care for unprivileged patients remains a challenge but progress is being made through collaboration between government, civil society and professional networks.

Cancer prevention should be a global priority. Additionally, improving cure rates is a pressing challenge. The establishment of healthcare systems capable of delivering multidisciplinary cancer therapeutics/care is essential in both LMICs and HICs. These systems play a crucial role in implementing evidence‐based cancer prevention, treatment and care, underscoring the importance of scientific rigour and continuous innovation in their work.

The African situation also underscores the broader issue of resource allocation, with many countries prioritizing military spending over health care, a concern that is not limited to Africa. The United States and EU are establishing quality‐assured CCCs for innovative cancer healthcare based on translational research. China is investing heavily in biological and technological research for prevention and cancer therapeutics/care. Australia reported a similar development at the previous Vatican conference [8].

The United States has long supported cancer research and care in LMICs. The Princess Margaret Global Cancer Program and the Stella Project (see Session 4) are additional initiatives that stand out. When launching the EU Mission on Cancer, the Commissioner Carlos Moedas emphasized ‘Open to the World’, signalling a commitment to global research collaboration. The Organisation of European Cancer Institutes (OECI), which oversees the EU's leading accreditation programme has also made a strong commitment to supporting African and South American cancer centres, instilling confidence in the future of cancer care.

Ultimately, global collaboration is crucial in addressing the growing cancer burden. Expanding partnerships and fostering innovative, multidisciplinary approaches for cancer prevention and therapeutics/care are urgent and essential steps toward reducing incidence and improving outcomes worldwide.

## 3 Session 2: The comprehensive cancer Centre (CCC) for innovative, high‐quality multidisciplinary cancer therapeutics/healthcare and healthcare‐dependent prevention, the fundamental infrastructure for translational cancer research

**Chair Alex Markham**, University of Leeds, United Kingdom

The following three sessions explored translational cancer research required to mitigate the cancer problem. Strategic analyses conducted 15–20 years ago led to the concept of personalized treatment, as per Lee Hood's P4 research concept, for major chronic diseases, including cancer [9]. While personalized cancer medicine was relabelled precision cancer medicine to reflect the strong focus on targeted anticancer agents, we propose a return to P4 Cancer Medicine. This approach, with its broader scope, includes Prediction based on both basic/preclinical research, reverse translational research, and patients' experiences, for Prevention, Personalized treatment and HRQoL research. Of critical importance, the latter must be Participatory, with the well‐being of the patient as the primary goal. This participatory nature ensures that all voices are heard and valued in the fight against cancer, making it a truly comprehensive approach.

With this background **Leroy Hood**, Phenome Health, Institute for Systems Biology, Seattle, USA, presented a keynote lecture with the title **The Synergy of Data‐Driven Health and Data‐Driven Informational Peptide Drug Discovery in Dealing with Data‐Driven Health and a Universal *N* = 1 Healthcare**.

Data‐driven health care employs a genome and longitudinal phenome analysis of each of many individuals to decipher their individual biological complexities and turn these insights into actionable possibilities to optimize wellness and healthy ageing, to avoid disease and to gain causal insights into wellness and disease. Two major additional factors influence each individual's health trajectory—their behaviour and their environment—and these influences can be monitored by longitudinal phenomics measurements. The actionable possibilities, so derived, will be processed through a knowledge graph to provide proper educational metadata for a transformer. Statistical analyses and machine learning analyses of individual genome and phenome data also lead to the discovery of many new possible drug candidates for wellness and prevention. These efforts will lead to an *N* = 1 healthcare of wellness and prevention.

For the candidate wellness and prevention drug targets, we plan to employ peptide drug discovery techniques that will utilize large‐scale DNA synthesis to generate millions to billions of 30‐mer peptide genes and then use the tools in cell and molecular biology to interrogate these drugs individually through phenotypic and targeted selection procedures. This approach will also map the peptide drugs' individual transcriptional activities by single‐cell analyses in 100 or more human cell lines and organoids and process each through a knowledge graph to generate the metadata used to instruct a transformer to model the optimization of drug use in individuals. These drugs will relate both to the optimization of wellness and aging and early detection of disease transitions and their reversal.

In addition, two AI‐driven approaches to healthcare were discussed: data‐driven health and informational peptide drug discovery and their AI‐driven synergies in moving to an *N* = 1 healthcare.

**Tit Albreht**, National Institute of Public Health, Slovenia, **Thomas Dubois**, French National Cancer Institute, Paris, France: **Structuring a Cancer‐Resilient Europe: the contribution of EUnetCCC**.

A common infrastructure to Connect, Elevate and Transform, launched in 2024 as the EU Network of CCCs (EUnetCCC).

Joint Action is Europe's strategic response to this challenge supported by a high‐level political decision after a long‐term strive of the cancer community for an EU‐wide structure that benefits society. It was laid out by the EBCP under Flagship 5 in 2021 and subsequently funded under the EU4Health programme. It brings together 163 partners across 31 countries with one goal: to establish a coherent, interoperable and certified network of CCCs that can serve as the operational backbone of Europe's cancer and health innovation agenda. EUnetCCC is built on four pillars:

**Equity**: ensuring all patients, regardless of geography, access to high‐quality care and research through a structured network of at least 100 CCCs across the EU — one per 3 to 5 million inhabitants, depending on the specific country context. EBCP clearly mandates the establishment of at least one CCC in all Member States regardless of their size, but not giving up on the criteria and standards for their establishment as CCCs.

**Excellence**: integrating care, research, prevention, education and innovation under common standards and governance, through a new EU‐wide certification framework aligned with European principles of transparency and non‐discrimination.

**Interoperability**: enabling mutual recognition of national and European designations and supporting emerging centres with a self‐assessment and maturity model.

**Solidarity**: fostering cooperation between leading and emerging CCCs, connecting them through digital platforms, shared infrastructures, and joint activities to build collective capacity.

This is not simply a labelling exercise. EUnetCCC aims to remodel CCCs into engines of territorial transformation, anchoring quality and innovation in national and regional cancer systems. By fostering the development of Comprehensive Cancer Care Networks (CCCNs), the project promotes cooperation both horizontally—between institutions within a shared network—and vertically—among healthcare professionals engaged throughout the patient journey. This integrated model ensures that quality, innovation, and research are no longer concentrated in isolated centres, but embedded into the broader continuum of cancer care across territories.

**Christian Brandts**, University Center, Frankfurt, and co‐spokesperson of the CCC network of the Deutsche Krebshilfe, Germany and **Simon Oberst**, Organisation of European Cancer Institutes (OECI), Brussels, Belgium, discussed **how to quality assure multidisciplinary cancer care in a defined geographic area around a CCC**.

Currently, there are almost 80 quality‐assured CCCs and large Cancer Centres in the EU, 14 in Germany in the programme of the Deutsche Krebshilfe, and 65 accredited by the OECI in 21 other Member States. The EU aims to incorporate many of these CCCs into a new network with EU financing.

However, the challenge remains of how to reach 90% of eligible cancer patients with quality‐assured cancer care. Present estimates of the coverage of both Cancer Centres and Networks around them range from 10% in some Member States to 90% in others, with the mean in the EU around 40% coverage. The broad definition of Comprehensive Cancer Networks was recently defined by the Cancon Joint Action [10]: they should be centred around a CCC, should embrace multiple care providers in a defined geography, and should include cancer research, education and training.

There is little literature on how to evaluate the effectiveness of Comprehensive Cancer Networks. The OECI has reviewed the international evidence and compared the results to its new set of Network Standards [11, 12]. From this evidence, OECI deduced 8 principles for effective Comprehensive Cancer Networks:

- The **governance** of the Network should be clear
- All Comprehensive Cancer Networks should have at least one **CCC** present
- The **patient pathways** in the network should be clear
- **Multidisciplinary team (MDT)** principles and structures should be the same
- The **strategic research** collaborations should be clear and promoted
- The **clinical guidelines** used by all centres should be the same
- There should be a consistent approach to the central **registration** of cancer patient data
- There should be **IT interoperability** and data sharing for MDTs (and into primary care) throughout the network

OECI has piloted certifications based upon the new standards in France and in the Netherlands. Examples of European countries which have developed a comprehensive range of cancer networks across the whole country include the Netherlands, Denmark, Finland, Ireland and Germany.

In Germany, Comprehensive Cancer Networks have been established to ensure outreach of and access to the CCCs' specialized services. The recent ONCOnnect initiative aims to strengthen and harmonize prevention/early detection, patient involvement, clinical trials activity, quality assurance and IT interoperability within the Comprehensive Cancer Networks.

Progress in Comprehensive Cancer Networks has the potential to create the necessary leverage to reach the highest standards of multidisciplinary cancer care.

**Anton Berns**, EACS and The Netherlands Cancer Institute, Amsterdam, The Netherlands, discussed **needs, criteria and quality assurance of CCCs** according to EACS' recommendations. Cancer Research Infrastructures are essential for enhancing cancer prevention, diagnosis, treatment and improving patients' quality of life. The creation of robust infrastructures across Europe—including registries, biobanks and data repositories—is key to strengthening the entire cancer research continuum. The EU's *Mission on Cancer* and *EBCP* promote such development, and CCCs are central to this effort. However, there are currently too few CCCs, and EU support remains limited and short‐term, despite the lengthy process required to establish high‐quality centres across all member states.

CCCs must go beyond providing state‐of‐the‐art care—they need to lead in basic, translational, and clinical cancer research, including areas like prevention, diagnostics, treatment innovation and quality‐of‐life studies. Equally important is their capacity to form alliances with local hospitals and other CCCs to broaden patient access and enable large‐scale clinical trials. These centres must integrate expertise across biology, pharmacology, diagnostics (including omics and AI‐assisted imaging), surgery, radiotherapy, immunotherapy and more. They also play a crucial role in training the next generation of cancer professionals.

Some CCCs may achieve excellence across multiple domains, earning a ‘Designation of Excellence’ from the EACS. However, all CCCs should be encouraged to develop at least one ‘Module of Excellence,’ which can uplift the entire institution by setting high standards and fostering cross‐departmental improvement.

Sustainability and long‐term investment are vital for building and maintaining such infrastructure, especially in underserved regions. The OECI has laid a solid foundation for CCCs, and the EU‐supported *EUnetCCC* Joint Action should build on this progress and closely engage the OECI to achieve the establishment of additional CCCs and promote networks thereof. To ensure equitable progress across Europe, a *European Cancer Agency*, modelled after the U.S. National Cancer Institute (NCI), could offer sustained funding, support network‐building, foster education and back high‐impact research from the bench to clinic and back.

To maintain quality, accreditation systems like those developed by the OECI and EACS can ensure high standards, while also enabling focused excellence modules within CCCs. Collaboration—both locally and internationally—is essential. *Cancer Core Europe* (CCE) exemplifies this by integrating seven CCCs into a unified network with shared trials, data systems, and regulatory frameworks. This has resulted in improved trial capacity, innovation, standardized practices, and researcher training, ultimately accelerating progress across Europe's cancer care landscape.

**Johannes Förner**, DKFZ Patient Advisory Council and NCT Patient Research Council, Germany, conveyed **opinions by a cancer patient advocacy representative on patient‐relevant excellence in cancer care**. CCCs play a central role in delivering multidisciplinary oncology care, advancing translational research, and fostering medical innovation. However, from a patient advocacy perspective, excellence in cancer care cannot be assessed by clinical outcomes alone. For patients, the experience of care is shaped by the ability to navigate complex systems, to be meaningfully involved in treatment decisions, to maintain quality of life during treatment, to have equitable access to innovation and to be structurally engaged in research and care design.

Many patients struggle to navigate the complex oncology healthcare system, especially those from disadvantaged backgrounds. A systematic review found that navigation programmes—run by professionals or trained laypeople—improve care by increasing adherence, speeding up diagnosis, enhancing coordination and sometimes reducing hospital readmissions, particularly for vulnerable groups [13].

Shared decision‐making (SDM) is another key aspect. The ‘SHARE TO CARE’ programme at University Medical Centre Schleswig‐Holstein, Germany, shows that SDM can be widely implemented, leading to greater patient involvement and satisfaction with treatment decisions [14].

HRQoL should be a central therapeutic goal. A large EORTC study found that patient‐reported outcomes like fatigue and appetite loss predict overall survival, highlighting their clinical relevance [15].

Despite universal healthcare, regional disparities persist. In the German federal state of Bavaria, only 16.9% of patients discussed in Molecular Tumour Boards come from rural areas, though these regions make up over 30% of the population. More regional and digital care models are needed [16].

The German National Centre for Tumour Diseases (NCT), a clinical research network of the DKFZ with CCCs, demonstrates how patients can be systematically involved in clinical–translational research, for example through the Patient Research Council (PatFoR), which helps design and prioritize early clinical trials.

In conclusion, the future of CCCs is promising, as it is moving towards a truly patient‐centred approach. This approach, which ensures access, guidance, shared decision‐making, and a focus on HRQoL, along with structural patient involvement, will serve as the pillars of innovation and excellence in the field of clinical research.

### 3.1 Conclusions and discussions

High‐quality cancer care requires multidisciplinarity and continuous innovation. CCCs integrate cancer treatment, healthcare‐related prevention, research and education, with the mission of delivering multidisciplinary care while driving innovation. They therefore represent the most important infrastructure for translational cancer research [17]. The United States has long been at the forefront, providing quality‐assured CCCs through an accreditation methodology established by the NCI.

The European cancer research analysis (2005–2007), initiated by Commissioner Philippe Busquin, laid the foundation for several European initiatives. France, Italy and Germany structured their national cancer programmes, and Germany introduced its own accreditation system for CCCs in 2008. The OECI launched a European accreditation programme in parallel with German Cancer Aid. Integration of healthcare, prevention, and research through the EU's initiatives, EBCP and the EU Mission on Cancer, represents a major step forward. The EU's current programme aims to increase the number of CCCs to at least 100, corresponding to one per 4.5 million inhabitants. Today, OECI has accredited 65 CCCs (including several in Italy and France), while German Cancer Aid has accredited 14 centres in Germany. With many additional centres already in the accreditation process, reaching 100 CCCs across the EU appears achievable.

Structuring translational research around CCCs presents another key challenge: reducing inequalities. Most patients are diagnosed and treated outside the catchment areas of CCCs. To address this, the EU project aims for 100 CCCs to cover 90% of cancer patients in the Union. This has spurred the development of Comprehensive Cancer Networks or Comprehensive Cancer Care Networks (CCCNs) to extend outreach from CCCs to surrounding regions. For these networks to ensure high‐quality care, they must be fully integrated with their CCCs. A minor problem is the complexity of nomenclature and overlap of abbreviations used in the strategic structuring of cancer care; for example, the abbreviation of Comprehensive Cancer Care is the same as that for Comprehensive Cancer Centre.

Another critical priority is patient involvement in translational research, particularly in the context of P4 Cancer Medicine. The challenge extends beyond ensuring consistent quality of care in CCC outreach areas to meaningfully including patients in research. In Germany, issues such as resource disparities and competition between providers, especially along the urban–rural divide, are being studied to enable inclusion in quality assurance by accreditation. Germany is actively working to guarantee broad access to advanced research infrastructures and to promote clinical data sharing.

There is also a need to quality‐assure advanced CCCs that possess the infrastructure and expertise for innovative translational research and effective collaboration. A programme for the Designation of CCCs of Excellence, initiated by EACS [18], has been proposed as part of the EU accreditation system. The latter could evolve into modules of excellence in specific areas of translational research and care, providing CCCs with aspirational pathways.

The overall objectives have strong support from patient organisations, particularly in Germany, where patients have been directly involved in planning nationally structured translational research within DKFZ and NCT.

A critical question remains: governance of CCCs. In many cases, responsibility for treatment and research is separated—both within Member States and across the EU. Common leadership is currently found in only a few European CCCs and the most advanced US centres. Consortia such as Cancer Core Europe and Cancer Prevention Europe have been established, making governance a priority for the future. The most complex challenge will be fostering effective collaboration and synergy among a large number of CCCs without creating an inhibitory bureaucracy. Here, the EU can learn from the NCI in the USA, helping pave the way for cross‐continental knowledge exchange.

## 4 Session 3: Translational cancer research, a coherent research continuum, is needed to innovate all clinical and prevention modalities and to achieve cost‐effectiveness

**Chair Alexander Eggermont**, Princess Maxima Centre for paediatric Oncology, Utrecht, Holland

Translational research is a prerequisite for mitigating the cancer problem, increasing the effectiveness of interventions and developing cost‐effective prevention and therapeutics/care. European analyses have revealed important gaps in implementing innovations and assessing clinical effectiveness as a basis for health economics analyses.

**Stefan Fröhling**, DKFZ, Heidelberg, Germany, presented **Developing Concept‐Changing Clinical Trials to Improve Cancer Care**. The National Center for Tumour Diseases (NCT) has evolved into a Germany‐wide engine for *concept‐changing* clinical research, closing the gap between laboratory discoveries and routine cancer care. Beginning with the two hubs in Heidelberg and Dresden—where translational laboratories, multidisciplinary clinics, and trial infrastructure have co‐existed since 2004 and 2015, respectively, the network is now expanding to six NCT sites encompassing 11 University Medical Centres and DKFZ. These highly specialized nodes are jointly supported by the DKFZ and partner university hospitals to deliver non‐commercial, science‐driven early‐phase clinical trials, ensuring fast and equitable patient access to innovation.

At the heart of this expansion lies the **Overarching Clinical‐Translational Trial Program (OCT)**, a single gateway through which investigator‐initiated trials are evaluated by an international board of more than 25 experts, including patient advocates, against criteria that span unmet clinical need, scientific rigour and patient‐centricity. Projects selected for OCT receive trial and infrastructure funding, coordinated regulatory support, and mandatory patient‐involvement frameworks, thereby accelerating translation while preserving academic independence.

An inaugural disease focus in the past years has been *rare cancers*, which account for nearly 25% of European cancer diagnoses, yet have a five‐year survival rate almost 15 percentage points lower than common malignancies. To date, NCT's precision oncology platform has molecularly profiled more than 5600 patients with advanced rare tumours, issuing genotype‐guided treatment recommendations in approximately 85% of cases and generating a disease‐control rate of about 55%. These insights underpin the upcoming RATIONALE trial, a multicentre, basket‐style diagnostic study (*n* = 946; launch Q2 2025) that tests whether molecularly guided therapy can double progression‐free survival compared to physician‐choice treatment. Currently the OCT2 program is rapidly widening its clinical trial portfolio into further priority research themes and disease areas.

From the outset, patients have helped co‐design study endpoints, outreach materials, and governance mechanisms, ensuring that research questions align with lived experience and that trial participation is both accessible and meaningful. By uniting comprehensive ‘omics’ diagnostics, rigorous trial methodology, and genuine patient partnership, the “One NCT” model aspires to revolutionise science into everyday oncology practice—first for rare cancers and ultimately for all patients who stand to benefit from concept‐changing clinical trials, instilling hope and inspiration in the field of oncology.

**Massimiliano di Pietro**, Early Cancer Institute, University of Cambridge, UK, discussed **Early detection—the potential roles for prevention and early treatment**. Gastro‐intestinal (GI) cancers account for about a quarter of all cancers globally and a third of all cancer‐related deaths, with approximately 5 million new cases each year. The majority of GI cancers develop in the context of pre‐malignant conditions. Early diagnosis of these conditions allows preventative measures or early treatment. For example, detection of blood in the stools by faecal immunohistochemical test (FIT) significantly reduces mortality by colorectal cancer via early diagnosis and treatment of colonic adenoma (polyps). Among GI cancers, oesophageal cancer has one of the worst prognoses with < 20% of patients diagnosed with it alive at 5 years. Oesophageal adenocarcinoma is the solid malignancy with the fastest rise in incidence in the Western World, with gastro‐oesophageal acid reflux and obesity being the main risk factors. We have developed at Cambridge University a pill on a string device (capsule sponge), which can be administered by a nurse in an office setting. Capsule sponge collects cells from the oesophagus and allows detection of the predisposing condition to oesophageal adenocarcinoma, also known as Barrett's oesophagus (BE). In a randomized controlled trial, we showed that offering a capsule sponge to people with acid reflux, increases by 10‐fold the rate of diagnosis of BE in the general population. We have also developed a risk prediction tool based on clinical and molecular biomarkers to stratify patients with BE based on their risk of early neoplasia or cancer. A national screening study is underway in the UK, recruiting 120 000 participants with history of acid reflux (males aged 55–79 and female aged 65–79) to determine whether capsule sponge screening can reduce mortality by oesophageal adenocarcinoma.

**Vanderlei S. Bagnato**, University of Sao Paulo, Brazil, Texas A&M University, USA, reported on **initiatives for affordable treatment of skin and cervical cancer in Brazil**. The problem of cancer treatment is not only a health issue but also involves economic and social aspects. New technologies have promoted significant advances in the treatment of the disease, but these advances remain outside the economic reality of many communities and are therefore inaccessible. It is certainly important to have technological developments in the field of cancer that incorporate a certain social responsibility, allowing broad accessibility. In this presentation, a case was reported carried out in Brazil regarding skin cancer and cervical lesions using principles of photodynamic therapy as the basis for development. Through the development of photodynamic therapy including both equipment and medications, it was possible to conduct a clinical trial involving more than 100 centres in Brazil and incorporating part of Latin America, where the treatment of non‐melanoma skin cancer was performed with new protocols and equipment, making the procedure easy and affordable. A success rate of approximately 95% complete tumour elimination with outpatient procedures was achieved. A training platform allowed training all professionals involved and collecting the results obtained. The recent development of soluble microneedles for the deposition of the photosensitive drug in the lesion will reduce patient preparation time and the total cost of the operation, achieving approximately 97% complete tumour elimination. Studies are being conducted with approximately 7000 patients, demonstrating that even long‐term follow‐up demonstrates that the procedures and treatment system implemented are as good as the best surgeries. The procedure has been approved by the Brazilian Unified Health System, which is expected to treat more than 80 000 new cancer cases with the new technology. Similarly to skin cancer, an initiative for the treatment of HPV‐type cervical lesions and carcinoma *in situ* has been implemented following the same path as skin cancer, and with sufficient success to be implemented in the future for the treatment of cervical lesions. The initiatives have led to the publication of around 200 scientific articles over the last 10 years and are intended to expand to other countries. More information can be found on the site: http://cepof.ifsc.usp.br.

**Richard Sullivan**, King's College, London, United Kingdom, analysed and presented **Treatment outcomes and new cancer medicines: better R&D for better social impact**. The last 20 years have witnessed relentless exponential innovation in the cancer medicines space, particularly targeted therapies and biologicals. Outside the Western biopharmaceutical ecosystem countries like China are also rapidly increasing their R&D in similar areas such as immuno‐oncology. Cancer medicines now dominate (> 85%) of biopharmaceutical pipelines. With this innovation has come significant challenges in delivering real value into cancer and health systems, that is best possible outcomes for lowest costs. Many new cancer medicines deliver significant clinically meaningful benefits. However, for example in immuno‐oncology, over 60% of drug‐indication pairs for solid adult cancers deliver moderate or very low clinical benefits suggesting a regulatory ecosystem that is out of alignment with the needs of society. The value consequences on countries are huge. In the English NHS alone, HTA authorized new cancer drugs, because of prices that are too high and clinical impacts for some drugs that are too low, have led to serious loss of net health benefits. For Europe, the situation is particularly acute because of the geopolitical turmoil induced by the current US administration leading to a contracting fiscal space. So what are the solutions to address? Innovation will continue but we must, as a collective solve the Baumol problem for cancer. Increasing technology and human resource costs but no improvements in productivity, in the face of a complex growing cancer burden. This requires a new paradigm in clinical trial designs and clinical research more broadly defined—shorter courses, lower doses, different delivery models, better quality—using the full range of transdisciplinary methods (economics and wider social sciences, operational science, HPSR etc). In addition, the currently accepted thresholds for marketing authorisation do need to be checked and challenged. In seeking to create headroom for downstream innovation health and cancer systems need to be re‐shaped through a more societally focused R&D agenda.

**Nils Wilking**, Karolinska Institute, Stockholm, Sweden, proposed that **real‐world data is required for implementation and research on clinical effectiveness and innovation in cancer care**. The rapid evolution of cancer therapeutics over recent decades has led to the approval of nearly 200 new cancer medicines by the European Medicines Agency (EMA) since 1995. However, the clinical effectiveness of these treatments is, in most cases, relatively unknown. This presentation highlights the critical role of real‐world data (RWD) and real‐world evidence (RWE) in bridging the gap between controlled clinical trial settings and the complexities of everyday clinical practice.

Traditional clinical trials, while rigorous, often focus on younger, healthier patient populations under ideal conditions. As a result, the derived data, although essential for initial regulatory approval, may not translate into effective or efficient treatment in broader, more heterogeneous populations. This discrepancy underscores the need for robust RWD, which is collected outside of experimental settings and reflects routine clinical care, including comorbidities, mixed HRQoL and treatment variability.

Nils Wilking examined the translational research continuum, from preclinical studies through early and late clinical trials to real‐world implementation and long‐term survivorship follow‐up. He stressed the importance of embedding outcomes research and health economics into this continuum to ensure that new treatments deliver value—not just efficacy. Particularly in oncology, where costs have dramatically increased (with cancer drug expenditures increasing from €12.9 billion in 2008 to €64.3 billion in 2023), the lack of meaningful postmarketing data creates major uncertainty in assessing cost‐effectiveness.

The presentation also addressed systemic challenges. The pharmaceutical industry's dominance in funding clinical research often leads to approvals based on limited evidence, typically small trials in non‐curative patient populations—without sufficient post‐market validation. Moreover, variations in national spending on cancer care (ranging from below €150 to over €400 per capita in Europe) highlight inequities and the need for data‐driven policy.

Emerging solutions such as Managed Entry Agreements (MEAs) and Coverage with Evidence Development (CED) offer frameworks for conditional access based on the collection of real‐life outcomes, ideally mitigating financial risk while generating valuable data. Tools like ESMO‐GROW and the upcoming European Health Data Space (EHDS), set for implementation between 2025 and 2031, are also discussed as pivotal developments in standardizing and facilitating the use of RWD across countries.

He concluded that, while oncology has witnessed remarkable therapeutic innovation, there remains a fundamental lack of systematic RWD collection and analysis. Addressing this gap is essential not only for ensuring the effectiveness and sustainability of cancer care but also for enabling more equitable access to treatment across Europe.

**Sara Faithful**, Trinity College, Dublin, Ireland, reviewed **Health‐related Quality of Life (HRQoL) and research**. HRQoL is a patient‐focused construct that provides data from the patient perspective of their, symptoms, physical function and emotional health. Patient‐reported outcomes (PROMs) are dimensions of HRQoL and are important in appraising treatment efficacy [19]. As survival differences between therapies are less distinct and populations more diverse, the need to personalise and optimise cancer treatment through patient participation becomes more important. Systematic monitoring of cancer patients' symptoms through PROs using the EORTC QLQ‐C30 has been shown to be an independent prognostic factor informing overall survival [20], this can inform proactive management and risk stratification. PRO measures used routinely can be a more accurate way to measure the impact of cancer treatment. Comparison of patient and physician‐rated toxicity scores consistently shows that observer‐rated toxicity underrepresents patients' own appraisal of symptoms with these differences leading to under reporting of treatment adverse effects [21, 22]. HRQoL measures increase the quality of information in studies and give greater understanding of treatment long‐term and late effects.

Population and cohort studies including HRQoL measures have advantages as they provide large‐scale information on cancer treatment effects, identify inequalities in health across populations and in differing healthcare settings. These studies can be used to obtain a broad perspective of cancer patient treatment outcomes and HRQoL at a single point in time and can inform health policy [23]. These data include more diverse populations than those studied in clinical trials, providing real‐world evidence that can form clinical practice [24].

HRQoL data collected as part of population and cohort studies can provide useful insights into rare cancers, difficult‐to‐reach populations and therapy outcomes. This knowledge is critical for informing long‐term follow‐up, new treatment strategies and healthcare interventions for late effects to improve outcomes for cancer survivors [25].

HRQoL measures and PROs used in routine clinical practice have been proven through multiple RCTs to provide significant patient benefits compared to routine care systems. Benefits include earlier symptom detection that facilitates earlier symptom management, better communication and treatment adherence [26]. These improvements in patient care led to reduced hospital stays and unplanned emergency admissions [27, 28]. PROMs used routinely have been shown to be a more accurate way to stratify patients according to risk and provide personalized and responsive supportive cancer care. Integrating ePROMs and HRQoL measures into routine clinical practice has been called for in transforming cancer services [19, 29]. However, despite this evidence and call for action implementation is variable across Europe. To improve and transform cancer services we need to value patient perspectives, invest in the technology and integrate PROMs and HRQoL into National Cancer Plans.

**Peter Lindgren**, The Swedish Institute for Health Economics and Karolinska Institutet, Stockholm. Sweden, presented **The cost of cancer and its implication for the research agenda**. The economic burden of cancer in Europe has surged over the past three decades, driven by shifts in healthcare delivery, demographics and technology. Healthcare costs have risen by 135%, while indirect costs, like lost productivity from premature death, have declined by 16%, indicating improved survivorship. Notably, the average cost per patient has stayed relatively stable, with the overall rise largely due to a 60% increase in diagnosed cases since 1995. This growth stems from population ageing, lifestyle risks and enhanced diagnostics. The share of Europeans aged 65+ rose from 15% to 21%, while obesity, alcohol use, and UV exposure have become more prevalent. Advances in screening and disease registration have also elevated reported case numbers, alongside longer lifespans that allow more individuals to develop cancer.

Cancer‐related healthcare spending varies across the EU from €130 in Hungary to €400 in Germany, averaging €268 per capita for the whole union. These discrepancies reflect differences in system capacity and access to innovation. Care delivery has shifted, with inpatient services declining in favour of outpatient treatments, indicating shorter stays and the adoption of less invasive therapies.

Pharmaceutical expenditures have grown substantially. Nearly 200 new cancer drugs were approved in the past 30 years, with the annual rate rising from one or two in the 1990s to about 14 today. Many enter the market with limited clinical evidence, prompting concerns about long‐term value. This highlights the need for robust real‐world data to monitor outcomes and inform cost‐effective use.

Preventable cancers, especially those linked to tobacco, still exert a heavy toll. Lung cancer exemplifies the potential for reducing incidence, deaths, and costs through targeted interventions. Prevention, if cost‐effective, is a powerful tool for both public health and financial sustainability.

Early diagnosis greatly affects outcomes and costs. Managing cancer in early stages, such as melanoma or kidney cancer, can save thousands per patient. Effective screening and early detection strategies can thus yield significant economic and clinical benefits.

Addressing cancers economic impact requires a comprehensive strategy. Prevention and screening are key but must be evidence‐based and cost‐effective. New treatments should be deployed efficiently, with real‐world monitoring. International collaboration is essential, especially for rare cancers. The future of cancer care must strike a balance between innovation and economic prudence to ensure long‐term viability and equity in access to care.

### 4.1 Conclusions on presentations and discussions

Future research strategies should focus more on prevention, cure, and health‐related quality of life (HRQoL) research, rather than converting cancer into a chronic disease. Translational cancer research is defined as a coherent continuum that spans from basic research to prevention and clinical applications, with a strong emphasis on long‐term follow‐up of outcomes (Fig. 1, 2). It should encompass all aspects of prevention (primary, secondary, and tertiary), screening programmes and therapeutics/care (diagnostics, surgery, radiation therapy, medical oncology, supportive care, rehabilitation, psycho‐oncology, nutrition, early and late palliative care and survivorship research).

Early translational research sets the agenda for this continuum, either through direct innovations from basic and preclinical research or indirectly via reverse translational research. As discussed in the previous Vatican Conference [8], strong interaction among basic, preclinical and clinical/prevention researchers is essential for developing new prevention contexts and therapeutic concepts. Examples include identifying cancer causes, refining cancer risk criteria, detecting precursors of invasive cancer, and improving the early detection of metastatic disease to enable timely treatment and HRQoL research. Achieving these goals requires new diagnostic technologies. For instance, experiences from gastrointestinal cancer illustrate the value of screening for detecting both invasive cancer and precursor lesions. Screening programmes initiated under EBCP can be used not only for prevention and early treatment but also for detecting disseminated disease at earlier stages.

Future treatment requires new and broader targets. Promising directions include immunological strategies and advanced stratification technologies for both tumour and patient characteristics. Next‐generation clinical trials should be structured more towards comparative trials and multimodal treatment, while implementation research must critically evaluate clinical efficacy and translate it into effectiveness in broader patient populations. Outcomes research based on RWD will be key to understanding the actual benefits of new anticancer agents, many of which are approved via Fast Track procedures with limited evidence of clinical value, often tested in patients with incurable disease at early palliative stages. Linking RWD collection with HRQoL research, including frailty scoring and systematic registration of side effects, may provide the robust evidence base needed to optimize therapies for these patients (about 10 million globally each year).

Health economics research is another critical but underdeveloped element. The overall European healthcare costs increased 135% to EUR 146 billion (1995–2023), largely unaffected by a 60% increase in incidence [30, 31]. The present use of many new anticancer agents creates an increasing economic burden for healthcare systems. European analyses show a continuous increase in direct health care costs, mainly driven by the costs of anticancer agents, which in 2018 represented a share of 31%. In the time interval 2018–2023, the sales of cancer medicines at list prices doubled to EUR 64.3 billion. However, due to undisclosed discounts, the costs may vary in different countries, regions and hospitals as the outcomes of negotiations are confidential. Consequently, the cost‐effectiveness of treatment with anticancer agents cannot be estimated despite effective outcomes research based on RWD and RWE. This global cost problem was also discussed at a previous Vatican Conference [8].

In contrast, research on photodynamic therapy for skin cancers and early cervical lesions offers an example of cost‐effective treatment, as demonstrated in Brazil in collaboration with other South American countries.

The rising prevalence of cancer, now affecting around 5% of the population in several countries, is another pressing challenge. It is driven by both an increase in chronic disease and longer survivorship among patients with long‐term side effects requiring continuous monitoring, additional treatments and supportive care. This reality underscores the need to focus on prevention and curative treatment, as well as on establishing evidence‐based guidelines for incurable patients, supported by HRQoL research.

## 5 Session 4: International collaboration, science progress and critical mass problem related to the development of P4 cancer medicine research

**Chair Anton Berns**, Netherlands Cancer Institute, Amsterdam, Holland

**Josep Tabernero**, Vall d'Hebron Institute of Oncology (VHIO), Barcelona, Spain, discussed **International collaboration between CCCs – Cancer Core Europe for clinical research**. Modern oncology is progressively breaking down silos and bridging the distance between scientific disciplines and geographical locations, embracing the integration of cancer research, clinical care and education. Key to realizing the vision of P4 Medicine is coordinating actions between institutions across European Member States. CCCs, sharing a commitment to multidisciplinary cancer research and care, are uniquely positioned to drive progress in this endeavour.

Cancer Core Europe (CCE) is a consortium of seven leading European CCCs that exemplifies how institutional collaboration can accelerate progress in precision oncology. Through its four pillars—clinical trials and translational research, education, virtual data centre and prevention and early detection‐ along with the transversal task forces, CCE fosters joint efforts to accelerate clinical innovation, create spaces for training and knowledge exchange, and build shared platforms for enhancing data sharing across borders. A key initiative of this joint effort is the cross‐institutional Molecular Tumour Board (MTB), which enables harmonized interpretation of molecular patient profiles and ensures equitable access to precision oncology strategies across centres. The MTB not only enhances clinical decision‐making but also strengthens data generation and knowledge exchange, which are essential components of participatory and personalised cancer care.

This effort is further illustrated by VHIO's participation in the CCE‐DART project, a CCE‐led initiative focused on designing data‐rich adaptive clinical trials. By integrating real‐world data, advanced analytics, and shared trial designs, CCE‐DART aims to make clinical research more efficient, informative and patient‐centred. These developments reflect a commitment to creating trial methodologies that are responsive to patient heterogeneity and evolving scientific knowledge, which represent critical elements of predictive and preventive oncology.

Supranational European initiatives are further reinforcing this collaborative drive. The CCI4EU initiative is making progress to develop, expand and interconnect CCCs as hubs of excellence and innovation across EU Member States. In parallel, the Joint Action on Comprehensive Cancer Centres (EUnetCCC), in which VHIO is actively engaged, is building a sustainable European network of CCCs aimed at reducing disparities, sharing standards and scaling best practices. Notably, the implementation and expansion of cross‐border MTBs is a strategic objective of EUnetCCC, that reflects its commitment to precision oncology as a strategic action for improving cancer care in Europe.

Collectively, these efforts illustrate how, through collaborative frameworks, shared learning and coordinated actions, European CCCs are paving the way towards a more personalized, participatory and impactful oncology ecosystem.

**Joachim Schüz**, International Agency for Research on Cancer (IARC), Lyon, France, presented **International collaboration aiming at cancer prevention—Cancer Prevention Europe (CPE)**. The case for cancer prevention in Europe, at a fundamental level, is the same as for all other parts of the world. The number of cancers is increasing, driven by demographic change and evolution in the exposure to risk factors, while the cost of treating patients is likewise spiralling. The growing cancer burden, spiralling costs for treatments, and the suffering from the treatment together with often adverse late effects, clearly underscore the need for primary cancer prevention.

However, the majority of cancer research investment continues to be made in basic science and clinical research, whereas investment in primary cancer prevention has been neglected, for manifold reasons. First, the discovery of the most successful primary cancer prevention is specific to cancer type and needs large resources for long‐term research for each distinct type, starting with the understanding of the causes, intervention research to identify the most effective prevention interventions and, finally, implementation research to identify how such effective interventions are to be implemented under local circumstances and available resources. Second, even if implementations have been compiled into national cancer control plans, the launching of respective campaigns remains a challenge, because the results are difficult to recognize in individuals and their impact may take several decades to emerge. Third, prevention campaigns for most cancer risk factors are only successful when there are synergies between individuals' compliance with prevention recommendations supported by society‐level or community prevention measures. Many unhealthy habits come from products that create revenue for a country's economy, so prevention is considered not only expensive but also a threat to economic growth, which is not true when balancing its related costs of treatment and rehabilitation from disease. It therefore needs strong ambassadors and visionary politicians to support cancer prevention and its implementation.

International collaboration is key to creating a critical mass of ambassadors advocating and assisting in the implementation of cancer prevention. Cancer Prevention Europe (CPE) originated from the general and collective recognition that cancer prevention in Europe is fragmented and lacks an overall strategy. The setup of CPE can be used as a blueprint for other regional networks for the same purpose, with its prevention strategies informed by IARC's World Code against Cancer. With globally more than 20 million newly diagnosed patients with cancer every year it is the 11^th^ hour to strengthen cancer prevention and international collaboration is a key element of it. For further reading [32, 33, 34].

*Where authors are identified as personnel of IARC/WHO, the authors alone are responsible for the views expressed in this article, and they do not necessarily represent the decisions, policy, or views of IARC/WHO*.

**Angelika Eggert**, Charité Comprehensive Cancer Centre, Berlin, Germany, reported on **Current Advancements and Challenges in Paediatric Oncology**. Recent advancements in paediatric oncology have significantly transformed the diagnosis, stratification and treatment of childhood cancers. Precision medicine programmes are at the forefront of this transformation, leveraging genomic and transcriptomic profiling to tailor therapies based on individual tumour characteristics. These initiatives have led to the identification of actionable mutations, enabling targeted therapies that improve outcomes while minimizing toxicity. However, access to targeted drugs and drug combinations is still a challenge for paediatric oncology. A potential lack of precision in bulk sequencing data and a new focus on tumour evolution are being addressed by innovative (spatial) single cell technologies applied to longitudinal tumour samples. Concurrently, liquid biopsy technologies—using circulating tumour DNA (ctDNA) and other biomarkers—are emerging as minimally invasive tools for early diagnosis, real‐time monitoring of treatment response, and detection of relapse. These assays are particularly valuable in paediatric settings, where repeated tissue biopsies pose substantial risks. In parallel, immunotherapies, including chimeric antigen receptor (CAR) T‐cell therapies, (bispecific) antibodies, and tumour vaccine strategies, are gaining traction in the treatment of refractory and relapsed paediatric malignancies. While challenges remain, such as treatment resistance, tumour evolution and plasticity, limited target antigens and immune‐related toxicities, early trials have demonstrated promising efficacy and durability of response. First evidence even suggests that at least some paediatric patients with acute lymphoblastic leukaemia can be cured with immunotherapy alone. Collectively, these innovations are reshaping the paediatric oncology landscape, with collaborative research efforts aiming to translate these breakthroughs into standard‐of‐care treatments and improve long‐term survival for children with cancer. The future use of PROMs in Europe‐wide cooperative clinical trials may help to further optimize treatment regimens to minimize acute and late adverse events.

**Danielle Rodin**, Department of Radiation Oncology, University of Toronto, presented **Advancing Cancer Research through International Collaboration: The Princess Margaret Global Cancer Program**. Advances in cancer research have led to improved methods of early detection, more targeted therapies, and a deeper understanding of tumour biology, offering new hope for personalized and effective treatments. However, there are major gaps in access to these innovations for populations most in need caused by socioeconomic and health system issues, affordability and availability of services, and discrimination and structural racism. As a hub for multidisciplinary expertise and research generation, CCCs play an important role in addressing these gaps. The impact of CCCs can also be heightened through collaboration with other international CCCs by offering greater opportunities to share best practices, advocate for resources, and increase the feasibility and scope of cancer research.

Princess Margaret (PM) Cancer Centre in Toronto, Canada, is the largest integrated cancer research, teaching and treatment centre in Canada. Its Global Cancer Program was launched in 2020 to provide infrastructure, resources, and coordination to collaborate with other CCCs in diverse practice settings. In October 2023, a consultation meeting was held in Toronto, which brought together the leadership of partner centres from HICs and LMICs. An important outcome of this meeting was the development of a network of institutions, which now actively collaborates in clinical innovation, quality, education, and research.

International collaborative cancer research that addresses issues of health equity and/or implementation science has been a pillar of the PM Global Cancer programme since its inception. Such international collaboration is essential to ensure that research addresses the heterogeneity of cancer across diverse patient populations and accrues sufficiently large study cohorts to generate meaningful, generalizable findings. Examples of ongoing projects include work on early detection involving screening and counselling for breast cancer genetic mutations (BCRA 1/2) in African women, an international multi‐centre radiation trial, and the deployment and evaluation of institution‐wide distress screening. These projects are all international in scope, pragmatic in design, inclusive of underrepresented populations, and engage patients and providers from diverse geographic and ethnic/racial groups.

In the last year, the Canadian Academy of Health Sciences released a report that renewed its commitment to Canada's work in global health. They identified academic medicine as an important mechanism for supporting research that addresses persistent disparities in outcomes, which have undermined the value of research dollars invested in therapeutic development. Networks of CCCs committed to collaborative research, with a strong focus on inclusivity and knowledge translation, are critical for achieving this potential.

**Manjit Dosanjh**, Project leader of STELLA, University of Oxford, United Kingdom and ICEC (International Cancer Expert Corps), Washington DC, reported on **STELLA: An initiative to overcome global inequalities in access to curative radiotherapy**. Radiation therapy (RT) is a critical tool for the treatment of cancer. Yet in the face of the growing burden of cancer diagnosis there is a global shortage in access to RT, especially in LMICs where 70% of cancer patients live [35, 36].

Modern radiotherapy is delivered using x‐ray beams by linear accelerators (linacs). 15 000 of these linacs are in service globally, yet linac‐based radiotherapy in LMICs is extremely under‐provisioned. Across Africa there is one radiotherapy device per 3.4 million of population, compared to 1 per 100 000 in the United States. Linac availability is hampered by ageing equipment, long lead times on spare parts and power shortages. This leads to continued reliance on dated Cobalt‐60 radiotherapy machines.

The STELLA (Smart Technologies to Extend Lives with Linear Accelerators) project is a novel, ground‐up redesign for a resilient and cost‐effective radiotherapy system tailored to challenging environments [37]. The project started in 2016 at CERN by the International Cancer Expert Corps (ICEC) in collaboration with the UK Science and Technology Facilities Council, the Universities of Cambridge, Oxford, and Lancaster and partners from all African countries.

Due to the shortfall of RT services in LMICs and the poor performance of current linac stock and the absence of detailed data regarding linac downtime and failure modes, a detailed survey was carried out in all the 28 African countries that had access to RT to examine the current status of technology and expertise [38, 39]. The results allowed the definition of the impact of design parameters of a future ‘STELLA’ facility that would function reliably with minimal operational and maintenance complexity in countries with challenging environments. It was the first major study of radiotherapy services in all African countries and included benchmarking against high‐income countries. Results showed a significantly longer mean downtime in African countries compared to HICs. The main factors for machine downtime were access to spare parts and lack of trained local staff able to repair and operate the machines [40]. Data from similar studies carried out in the Balkans and former Soviet Union countries showed the same underlying issues [41]. STELLA offers robust mechanical designs and tight interaction with AI technologies for failure prediction and workflow automation, as a solution to the problems faced in LMICs.

**Péter Nagy**, National Institute of Oncology, Budapest, Hungary, reported on **Innovation in Central‐Eastern Europe: The Main Gap in pan‐European Cancer Research inequalities** Besides socioeconomic disparities, exposures to risk factors and access to modern diagnostic and treatment options, inequalities in cancer research are also associated with the apparent differences in cancer mortality within Europe and worldwide. Sadly, only a small portion of the published scientific results is from the 13 member states that joined the EU in or after 2004, which largely represents the Central and Eastern (C&E) European region. Among others the OECI A&D programme and the 4UNCAN.EU coordination and support action demonstrated that although Europe is a major leader in cancer discovery science, it is not reflected in the valorisation of the results. This is largely related to the immaturity of the ‘value chain’ in many member states, an area which is especially underdeveloped in C&E European cancer centres. Twinning (a concept that was formulated at the 1^st^ Vatican congress) proved to be an effective outreach mechanism to address inequalities and create stronger synergies between C&E and Western‐European cancer centres. Notwithstanding, there are still no dedicated funds within the large budgets of EBCP or the Cancer Mission allocated to twinning activities, to improve technology transfer or to address inequalities within cancer research and innovation by providing regional funds for game‐changing science. The second part of the presentation used the Hungarian example to show how strategic actions of a member state to support innovation can shape the cancer research and innovation ecosystem to provide practical results for the benefit of patients. Most importantly, substantial funding for large consortial research programs provided grounds to create synergies between clinicians and basic scientists and a more problem‐oriented innovative approach in translational cancer research.

**Manuel Heitor**, University of Lisbon, Portugal, discussed **Mission‐Oriented Cancer Research in Europe: towards a new policy framework to improve cancer care and research in the next decade**. The EU Cancer Mission, with its potential to significantly impact cancer research and care in Europe, could usher in a paradigm shift that is not only effective but also truly transformative. This transformative nature of the Mission is inspiring and should motivate all stakeholders involved to adopt a *paradigm shift* in cancer research and care towards effectively decreasing the number of cancer deaths in the coming decade. It requires that the current ‘narrative’ considering cancer as a ‘chronic disease’ is replaced by a new policy framework and investment in prevention, readiness and early detection.

This imposes new challenges for policymaking, including the effective implementation of *translational research* under a ‘coherent cancer research continuum’, which requires a new centrality and the effective institutional engagement of competitively selected research performers, notably ***Comprehensive Cancer Centres, CCCs***.

In addition, we argue that ***political ownership*** of the EU Cancer Mission should be effectively assumed at the level of an EC Vice President, who should guarantee the full integration of the EU Cancer Mission and EBCP. This should be implemented together with appropriate engagement and responsibility of the respective DGs and national authorities at Member States level.

Our evidence is built on strong European foundations in biomedical science and infrastructures that span from repositories to bioinformatics. Lessons learned from the attempt to promote *Mission Oriented Innovation Policy* (MOIP) in Europe, notably in cancer, show the need to guarantee the implementation of three main foreseen changes in Europe for the coming decade:

- the **priority given to *prevention, preparedness and readiness***, requiring that a fully revisited *Europe's Cancer Mission* integrated with *EBCP* is totally articulated and integrated in the interface of ‘The Health Union’, ‘The Preparedness Union’ and “The Skills Union”. The ultimate goal should guarantee implementing in Europe ***Predictive, Preventive, Personalized, and Participatory Cancer Medicine (P4CM)*** over the next decade;
- the need to better **engage young generations**, providing better jobs to guarantee a better future for them, together with ***Choose Europe*** to foster research careers in Europe. This requires that a fully revisited Cancer mission is totally articulated and integrated with ‘Choose Europe’ and MSCA (Marie Sklodowska‐Curie Action), as well as with ERC; and
- the need to **take much more risks** by accepting failures as steps to success. This requires that a fully revisited Cancer mission is totally articulated with an ‘Experimental Unit’ at EIC to implement new assessment and funding methods for cancer research and care in Europe.

Our analysis focuses on a critical assessment of the initial 4 years of Horizon Europe, 2021–2024, to help promote Europeans' wellbeing and improve the way cancer care and research will be funded and governed in Europe during the *Multiannual Financial Framework* 2028–2034 to effectively decrease the number of cancer deaths in Europe in the coming decade.

**Bengt Jönsson**, Stockholm School of Economics, Sweden, presented a critical review: **How is cancer research funding adapted to the goals of translational research to achieve evidence‐based medicine and cost‐effectiveness?** Very small resources are devoted to late translational research. This is a growing problem with the fast introduction of new cancer medicines with inadequate data for implementation in healthcare systems, and the high and growing costs for new cancer medicines for healthcare systems.

Global public and philanthropic funding of cancer research has doubled in current prices between 2005 and 2024, while adjusted for inflation the increase has been very small, both in Europe and the United States. In the United States funding is concentrated in NCI and NIH, while in Europe funding is scattered between many countries and philanthropic funders. EU funding is despite a significant increase relatively small.

Total R&D spending in the pharma industry has increased three times between 2005 and 2024 and the share devoted to cancer has increased from just below 10% to recently between 30 and 40%. Global sales of cancer medicines have increased 10 times since 2005 to about 250 billion USD in 2024.

Public funding for cancer research has nearly doubled in current prices between 2005 and 2024, but R&D spending for cancer research in the pharmaceutical industry has increased 10 times. In 2005 public funding was twice that of private industry funding, but in 2024 private funding was twice that of the public one.

Three quarters of all public/charity funding awards for cancer research are for preclinical research, 10% for public health, 7% for clinical trials, 3% for radiology and 1% for surgery.

Available data shows that a great unbalance between public and private for‐profit funding has occurred during the last 20 years. Public funding has changed from twice the private for‐profit to half between 2005 and 2024. Public funding for late translational research is only a fraction of pharma industry R&D funding and only 1% of public spending on cancer medicines.

Public and philanthropic funding, mainly focused on basic research and development of new medicines, has been constant in fixed prices over the last 20 years. Private for‐profit research in the pharmaceutical industry, mainly for clinical trials in the commercialization phase, has increased 6‐fold in fixed prices. Increased public funding for late translational research is needed to inform clinical practice where costs can be reduced and outcomes improved thus improving efficiency and equity in cancer care.

**Nancy Abou‐Zeid**, Fondation ARC, Paris, France, informed about the **FORCE Consortium – Fostering Oncology Research by Charities in Europe**. Cancer charities in Europe play a crucial—but surprisingly underestimated—role in the research landscape. Working together with patients and citizens, charities respond to patients' priorities by supporting high‐quality research. Pragmatic clinical trials are of particular interest in this context. Indeed, these trials focus on improving treatments and optimizing therapeutic strategies while providing adequate, accessible, and affordable short‐term therapeutic solutions that will benefit as many patients as possible.

The FORCE consortium is funded by the EU Cancer Mission under the Horizon Europe Framework Programme for Research and Innovation (2021–2027). Coordinated by Fondation ARC (France), it is composed of **14 charities representing 11 countries**. The FORCE consortium aims at developing and implementing an **ambitious, yet feasible, programme by and for cancer charities**.

This **Pragmatic Clinical Trial Programme will be designed to tackle high and unmet medical needs in oncology** with a focus on hard‐to‐treat or rare cancers. Through the successful achievement of its objectives, FORCE will strengthen cooperation among European cancer charities and mobilise the European research community to **improve health outcomes for cancer patients through patient‐focused research**.

**The overall objectives of FORCE are:**

1. Define and deliver a Pragmatic Clinical Trial Programme by cancer charities for rare and hard‐to‐treat cancers.
2. Implement two transnational calls for proposals to select and fund pragmatic clinical trials.
3. Deliver to the European Commission a blueprint on ‘pragmatic oncology clinical trials funding by charities’ to facilitate decision‐making.

The Pragmatic Clinical Trial Programme will be elaborated based on **a bottom‐up coordinated approach**, involving all relevant stakeholders across Europe: researchers, clinicians, professionals from different disciplines, European CCC representatives, patients and patient organizations, cancer survivors, citizens, policy makers, regulatory authorities, European charities, etc.

Specifically, FORCE aims at reaching a working definition of what constitutes a **pragmatic clinical trial suitable for European cancer charities**' **support**. A special focus will be made on defining precisely in which way pragmatism can maximize patient impact and determining the types of pragmatic trials to be supported by charities. The research priorities will be set based on unmet medical needs but also on patients' perspectives and needs. FORCE will also interact with relevant European initiatives, in particular those related to **data sharing** in order to optimize research acceleration in the benefit of patients.

**Ingemar Ernberg**, Karolinska Institute, Stockholm, Sweden, discussed **the ultimate need for improving and coordinating education in developing P4 Cancer Medicine**.

The implementation of P4 Cancer Medicine into applied cancer healthcare ultimately will depend on education and dissemination at multiple levels of the academic and healthcare systems. This will require an effort not yet seen to improve the education, its contents, focus and organization. The challenge is demanding, as we are dealing with education at many levels and provided by different stake holders which have so far not been interacting with each other to improve curricula and increase efficiency by the right focus and decreased overlaps. There is no overall coordination to streamline the education and dissemination from that of the undergraduate medical profession to the general public, patients and their next of kins. We had suggested that an analysis of the current situation should have been part of the ongoing Mission on Cancer and the EBCP, in an effort to bring together the stakeholders at different levels and form a future strategy to improve the continuum of education. Instead, different calls have included demands on non‐coordinated educational work packages as a homage to ‘we must have some education also’. This has just added to the fragmentation.

Currently the undergraduate training is provided by medical faculties and medical or nursing schools, the skills of professional specialization by the healthcare system, the professional academic development by the healthcare system, universities and external providers like the healthcare authorities or even private providers, the post‐specialization life‐long continuous upgrading of knowledge and skills by specialist associations and international societies, for nurses often by the healthcare providers, academic postgraduate training by universities and the knowledge dissemination to the patients and the general public by cancer societies, patient organizations, and public authorities. Each one of these important actors operates within its own bubble with limited or no communication or coordination with the others.

### 5.1 Conclusions on presentations and discussions

The increasing complexity of cancer biology, the growing need for translational cancer research, and the demand for supporting infrastructures require strong collaborations between quality‐assured CCCs. The efforts by the EACS and other stakeholders through the EU‐funded EUROCANPLATFORM project to establish both Cancer Core Europe (CCE) and Cancer Prevention Europe, contributed to the European Commission (EC) launching the Mission on Cancer. This represents a significant step that highlights the importance of the EC's role in advancing cancer research.

Forming consortia of CCCs is a European achievement and remains a challenge in the USA. Access to patients and biological materials, combined with the sharing of advanced technological resources, is a prerequisite for further innovation in translational research. Paediatric oncology has already demonstrated the value of collaboration across clinics to address limited patient numbers, but it still requires the support of CCCs to expand advanced translational cancer research. Several fields, such as surgery, radiation therapy, medical oncology (including targeted drugs and immunotherapy), geriatric oncology, outcomes research based on RWD, and HRQoL research depend on collaboration across centres to reach the critical mass needed for innovation.

Closer collaboration between centres in less‐developed research environments and CCCs in HICS, often referred to as ‘twinning’, can create mutual benefits. In large countries, the urgency of national collaboration among CCCs cannot be overstated; Germany provides a successful example. Nationally structured translational research also facilitates international partnerships, such as the Princess Margaret Global Cancer Program, which serves as a leading example. Another initiative, the Stella Project, is helping countries with limited resources develop radiation therapy infrastructures.

For mission‐oriented cancer research in the EU to succeed, a critical evaluation of progress is essential. Current implementation of strategies proposed by the EACS and the cancer community remains fragmented, and available economic support is not aligned with the programme's goals. Funding is a major obstacle: the EU's direct contributions are modest, public funding in Member States is fragmented, long‐term programmes often lack sustained support, and for‐profit organisations, particularly the pharmaceutical industry, play an increasingly dominant role. The overall strategy focuses on the development of cancer into a chronic disease. Since 2005, not‐for‐profit funding has doubled (excluding inflation), while for‐profit funding has increased tenfold and now supports most clinical cancer research. This dominance not only complicates the work of charities that aim to collaborate in academic translational research but also raises concerns about the potential impact on the quality and affordability of cancer care.

Education is another critical issue, currently hindered by fragmentation. Cancer Core Europe, with support from the EACS, established a summer school for translational cancer research in 2012, which has become an important initiative. However, a broader expansion of education is needed urgently. This requires not just financial support, but also better coordination and stronger financial support from not‐for‐profit funding bodies, rather than the current reliance on the pharmaceutical industry.

## 6 Session 5: Development of international cancer research and comparative assessments—viewpoints on initiatives from different countries and regions

**Chairs Manuel Heitor**, University of Lisbon, Portugal, and **Chien‐Jen Chen**, Academia Sinica, Taipei, Taiwan

What are the next steps in the development of translational cancer research aiming at evidence‐based and cost‐effective P4CM? Which strategies should be prioritized? What are the opportunities to expand international research collaboration?

## 7 Overall discussion and conclusions for a conference statement

**Chairs Joachim von Braun and Michael Baumann**. The statement is published also separately at the PAS website.

https://www.pas.va/en/events/2025/cancer_research/final_statement.html [42].

### 7.1 Statement of the conference

#### 7.1.1 Abstract

This conference explored strategies to structure translational cancer research in order to increase its effectiveness, innovation and reduce global inequalities. Despite major advances in cancer biology that have reduced mortality in high‐income countries, significant disparities persist across and within countries, driven by disparities in care, as well as systemic and socioeconomic barriers, and the fact that most patients still die from their disease. The conference emphasized the need for a fully integrated research continuum—linking basic science, clinical research to application, and real‐world outcomes—to make more scientific progress and ensure all patients benefit from this progress. Key priorities include expanding Predictive, Preventive, Personalized, and Participatory Cancer Medicine (P4CM); strengthening infrastructures; and most importantly Comprehensive Cancer Centres (CCCs); addressing personal data‐driven health, digital health and AI opportunities while continuing to strongly support more traditional areas of cancer research; and embedding health economics and implementation science. A shift towards publicly funded, mission‐driven research was strongly recommended to counterbalance commercial dominance, align cancer innovation with quality improvement and equity goals, and because prevention and screening research does not usually attract commercial support. The importance of international collaboration, data interoperability, early detection/intervention and culturally tailored care was underscored, with a strong emphasis on data interoperability to ensure the efficiency and effectiveness of cancer research. By investing in prevention, early detection and interception, quality of life, and equitable access, the global cancer research agenda can become a model of solidarity, sustainability, and ethical responsibility.

#### 7.1.2 Purpose and findings

##### 7.1.2.1 Purpose of the conference

The increasing knowledge of cancer biology and technical progress in the last few decades has set the stage for more effective translational research, covering all therapeutics/care and prevention strategies. This conference aimed to analyse and discuss how to structure cancer research to continue to make progress against this disease and how its early detection/intervention and optimization of treatment can at the same time reach all patients across geographic regions, reduce inequalities, and ensure access to the critical mass of patients and biological information required for translational cancer research. The overarching goal is a long‐term strategy that simultaneously increases innovation and reduces inequality, through evidence‐based and cost‐effective prevention and therapeutics/care, ensuring improved quality of life and equitable outcomes for all cancer patients.

##### 7.1.2.2 Uneven Progress and global cancer burden

While we recognize significant scientific progress, its distribution remains highly uneven—between global north and south, and within individual nations and regions. Cancer continues to escalate as a global health crisis with currently about 20 million new cases and about 10 million deaths annually. Disparities in care and outcomes, driven by socioeconomic and systemic inequalities, persist. A core finding is the opportunity for global learning and action in prevention, diagnostics, cure, vaccination and research funding to close these gaps.

##### 7.1.2.3 A multi‐actor task for science, innovation and moral responsibility

The scale and complexity of cancer demand collective action across sectors: from basic and clinical scientists to policymakers, healthcare providers, patients, patient advocates, and religious and moral communities. Religious communities, in particular, can play a significant role in moral discourse, providing a voice for the poor and excluded, and fostering ethical frameworks for action. It is in this collaborative spirit that they can foster ethical frameworks for action, ensuring that everyone is engaged and involved in the process.

##### 7.1.2.4 Digital health, data and AI opportunities

The transformation into data‐driven cancer medicine is underway. The integration of artificial intelligence and digital platforms across the cancer continuum—from digital pathology to remote tumour boards—offers vast potential, while also recognizing that traditional areas of cancer research need to continue to be strongly supported. However, challenges like digital inequity, fragmented systems, security and fair access, and underinvestment must be addressed. A major priority is building infrastructure for interoperability, biobanking, omics technologies, and real‐world data integration. Registry‐based randomised clinical trials (R‐RCTs) and patient‐reported outcome measures (PROMs) must be scaled.

##### 7.1.2.5 Translational cancer research and the P4CM model

Basic science is the driving force behind impactful translational research. Translational cancer research must span the full continuum: from preclinical disease insights and early diagnosis to implementation and outcomes research. We emphasize the development of Predictive, Preventive, Personalized, and Participatory Cancer Medicine (P4CM) as a strategic framework. Efforts should include early diagnosis /intervention, reverse translation, liquid biopsy technologies, development of technologies which can be utilized world‐wide, and data science‐based innovations. The key goals include reducing cancer incidence and early detection and reversal that also reduces mortality, increasing cure rates, extending survival, and improving health‐related quality of life. Prevention remains underfunded despite its potential to reduce incidence dramatically. The same applies for early detection which has significant potential to increase cures rates with treatments available today. Cancer is a complex disease that perturbs multiple normal systems (e.g. angiogenesis, metabolism, inflammation, etc.) and in the future targeted multimodal therapies are going to become an essential approach for cures. Quality of life and palliative care research need much greater visibility and economic support. To bridge the growing gap between innovation and real‐world delivery, it is crucial to focus on operational and implementation science, which can help translate research findings into practical applications.

##### 7.1.2.6 Comprehensive cancer Centres (CCCs) as structural backbone

CCCs are pivotal to organizing research, care and education. They provide a structural model for excellence across the cancer continuum, integrating high‐quality care with research and outcomes measurement. The expansion of CCCs and their accreditation (such as OECI, CCCoE) is crucial, especially in the context of the EU‐supported goals of increasing CCCs from ~50 to ~100. Expansion of CCCs must continue. EU‐supported goals of increasing CCCs from ~50 to ~100 are crucial. Collaboration across CCCs—including twinning programs and coordination with regional hospitals—is vital to ensure every patient benefits from scientific progress. National platforms like Germany's DKTK and “One NCT” with its OCT clinical trial program exemplify seamless pipelines from lab to bedside, with international review, fast‐track implementation, and strong patient involvement. Internationally expanding consortia of CCCs are represented by Cancer Core Europe and Cancer Prevention Europe. These should be expanded globally whenever they fit to the national or regional structures of health care organization and promise better access of all citizens to optimal care.

##### 7.1.2.7 Equity and global disparities

Inequities remain a central issue in cancer care. Efforts must include strengthening primary health systems, decentralizing services, expanding insurance literacy, and training the next generation of researchers and health care providers. Globally, HPV, HBV, and HCV‐related cancers—especially in Africa—require urgent action through vaccination, screening, antivirals and policy reform. Europe should lead, for example, to ensure equitable access across all member states and supporting regions with unmet needs collaborative networks. Also, in translational cancer research great inequalities exist, with negative consequences for developing modern integrated research/care systems.

##### 7.1.2.8 Funding models and economic challenges

The current funding environment does not adequately support the full translational research continuum. Most funding comes from for‐profit entities, particularly the pharmaceutical industry, which limits academic agenda‐setting and limits research oriented towards prevention and screening. While public investment has doubled since 2005, for‐profit funding has increased tenfold, now accounting for ~80% of total global cancer research spending. We call for a shift towards mission‐driven public and philanthropic funding models that prioritize equity and translational outcomes over short‐term commercial returns.

##### 7.1.2.9 Critical mass and international collaboration

The complexity and fragmentation of cancer biology and patient diversity require large‐scale, collaborative efforts. No single institution can independently sustain the research needed for stratified, personalized care. International and global networks must coordinate clinical trials, infrastructure development and digital tools like robotic diagnostics tools. At hemispheric levels, such as for Europe and the other mega regions, regional Cancer Institutes with a coordinating mission, bringing together current and emerging research and care institutions, and sustainable funding are essential.

#### 7.1.3 Research agenda and priorities

**Advance predictive, preventive, personalized, and participatory (P4) cancer medicine:**

- Early prediction/intervention (when the cancer perturbations are less complex), predictive risk factor modelling and omics‐based stratification and early detection.
- Personalized medicine, surgery and radiation based on molecular and imaging diagnostics.
- Combination therapies and immunotherapies for early and advanced disease stages.
- Real‐world evaluation of balanced anti‐tumour and supportive care approaches.
- Multimodal therapeutic approaches to cancer to deal with this entity as complex multifaceted diseases.

**Accelerate prevention research and support implementation research:**

- Focus on individual as well as population‐based screening strategies and vaccine development.
- Implement digital health interventions to scale access and reduce disparities.
- Strengthen implementation to widely and equitably disseminate current and future standard of care.

**Embed health economics and outcomes research:**

- Evaluate cost‐effectiveness from early‐stage development.
- Standardize the use of PROMs for all patient pathways.

**Infrastructure development:**

- Scale and accredit CCCs across all regions to improve access of patients to high quality care.
- Establish CCCs of Excellence with advanced data science, omics, and AI capabilities.
- Foster interaction with basic/preclinical centres and regional hospitals.

**Ensure equity:**

- Conduct systematic research on geographic and socioeconomic disparities.
- Promote culturally tailored care and prevention.
- Lower costs and increase access to key diagnostics and treatments in low‐resource settings.

#### 7.1.4 Recommended actions

1. **Address the challenges of universal cancer equity to improve the chances of longer quality survival time**
  - Sustain Curiosity‐Driven Basic Research and support fundamental research that seeds future breakthroughs.
  - Address the fact that access to quality cancer treatment is the privilege of those who can afford private health insurance.
  - Integrate underserved populations in all clinical and prevention trials.
  - Promote operational and implementation science to ensure delivery.
2. **Establish a Fully Integrated Translational Research Continuum**
  - Increase government spending on comprehensive cancer treatment and research.
  - Fund broadly cancer‐related basic science, translational projects and investigator‐initiated trials.
  - Support reverse translation, biomarker discovery and digital tool integration (AI, PROMs).
  - Focus resources on early detection/intervention of cancers.
  - Emphasize the power of multimodal therapeutic approaches to cancers as a complex disease.
3. **Strengthen CCC Networks and National‐Regional Platforms**
  - Increase the number and regional reach of accredited CCCs.
  - Promote models like ‘One NCT’ and international networks of CCCs to ensure rapid translation.
4. **Advance Implementation and Health Economics Research**
  - Fund real‐world outcome research and cost‐effectiveness assessments.
  - Embed economic evaluation in all phases of translational research.
5. **Promote European and Global Consortia**
  - Foster global collaboration on trials, resource‐sharing, and technology access.
  - Develop secure, interoperable platforms for cross‐border patient data and diagnostics.
6. **Create Enabling Policy and Funding Frameworks**
  - Shift funding balance towards public and not‐for‐profit institutions.
  - Establish mission‐driven research agendas with dedicated EU coordination.
  - Encourage philanthropic investment in equity‐based cancer research.
7. **Embed Education, Patient‐Centricity and Advocacy**
  - Improve awareness raising and education on cancer.
  - Build education modules within CCCs for next‐generation scientists and clinicians.
  - Empower patients as research co‐creators in governance and design.
  - Expand the use of PROMs and their integration into clinical workflows.

## 8 Conclusion

We call for a cohesive, equitable and innovative cancer research and care agenda—anchored in basic and translational science, global solidarity and ethical commitment. By embedding prevention, outcomes, health‐related quality of life and health economics research at the core, we aim to reshape the cancer landscape globally. Achieving this will require sustainable funding, structural reform and a shared vision that unites us all in our commitment to the dignity and well‐being of every patient.

## Conflict of interest

The authors declare no conflict of interest. Where authors are identified as personnel of IARC/WHO, the authors alone are responsible for the views expressed in this article, and they do not necessarily represent the decisions, policy, or views of IARC/WHO. Harold Varmus is the chair of WHO's Science Council. He has also been the chair of the Grand Challenges in Global Health initiative by the Bill and Melinda Gates Foundation.

## Author contributions

All authors drafted the abstracts summarising their individual talks, which were included in the report. All authors apart from WJ, SL, JM and CW attended the conference. UR, JC, and AB conceived the structure and provided project oversight for the manuscript. MB, JB, ML, AM, A Eggermont, AB, MH and C‐JC chaired the sessions. KR and PT contributed to discussions and conclusions. UR, JC, AB, MB and JB were responsible for synthesising and concluding the content of the different meeting sessions. UR, JC and AB wrote the original draft of the manuscript and involved MB and JB. All authors read, critically revised and approved the final manuscript.
